# Utility over novelty: How performance expectancy converts hedonic motivation, future expectations, and price sensitivity into private e-scooter purchase intention

**DOI:** 10.1371/journal.pone.0341194

**Published:** 2026-07-06

**Authors:** İbrahim Kırçova, Munise Hayrun Sağlam, Ebru Enginkaya, Akın Konuk

**Affiliations:** Department of Business Administration, Yildiz Technical University, Istanbul, Türkiye; Kaplan Business School of Australia, AUSTRALIA

## Abstract

This study explains private e-scooter purchase intention through a mechanism-based process model that links value cues to performance beliefs and, subsequently, to motivational readiness to commit. We argue that hedonic motivation, forward-looking expectations about the technology, and lower price sensitivity strengthen perceived performance (usefulness, reliability, and access). In turn, perceived performance primarily increases purchase intention by enhancing perceived autonomy and competence, while consumer innovativeness plays a limited role in translating beliefs into purchase intention. We employ a sequential explanatory mixed-methods design. Survey data (n = 338) are analyzed using partial least squares structural equation modeling, followed by 26 semi-structured interviews to interpret the pathways and identify boundary conditions. The findings show that perceived performance is the key appraisal in the model. It is shaped by enjoyment, future expectations, and price evaluation, and it influences purchase intention mainly through autonomy and competence rather than novelty-oriented exploration. The qualitative findings show that infrastructure, safety, parking clarity, service continuity, and transparency in ownership cost shape the strength of this mechanism**.** Overall, the findings support a utility-first adoption logic. For managers addressing younger urban consumers in emerging-market contexts, the priority is to make performance credibility and ownership support visible through reliable product performance and robust after-sales assurance. For policymakers in similar settings, protected links and more transparent governance can stabilize perceived performance and strengthen confidence in everyday use..

## 1. Introduction

Urban mobility is rapidly shifting toward electric, point-to-point modes. In North America, shared micromobility trips rose to 225 million in 2024 (+31% year-over-year) across 415 cities, and 74% of riders reported using shared bikes and scooters to connect with transit [1]. In Europe, 40% to 63% of riders use public transport, underscoring micromobility’s role as a feeder that expands network catchment areas and strengthens door-to-door connectivity [2] Among Micro-Mobility for Europe members, usage in 2024 exceeded 312 million e-scooter trips and 79 million e-bike trips [3]. For operators and city managers, this scale and intermodality create meaningful opportunities for ridership growth, integration, and service innovation while simultaneously elevating exposure to risks related to price sensitivity, safety, service quality, and regulation. These dynamics sharpen the need for management-oriented evidence on adoption drivers, willingness to pay, and service design choices that can sustain demand and improve system integration.

Regulatory and business-model headwinds have intensified alongside this growth. Paris removed rental e-scooters following a citywide referendum in April 2023, with the ban taking effect on 1 September 2023 [4]. Italy adopted stricter national rules in 2024, introducing mandatory helmet, insurance, and number plate requirements [5]. Several operators have restructured, most visibly Bird, which filed for Chapter 11 in December 2023 and re-emerged as a private company in April 2024 [6]. These developments underscore that market viability depends not only on ridership but also on unit economics, pricing architecture, and alignment between service operations and public governance. In practical terms, consumers’ mobility choices are increasingly made within environments where availability, cost, and perceived risk can change quickly, elevating the importance of understanding what drives demand and how value is formed.

Against this backdrop, an important question remains insufficiently explained: how do consumers form purchase intention for a private e-scooter? This question differs from shared-use adoption because private purchase requires a stronger commitment [7,8] and forces consumers to judge whether the product will be useful, reliable, safe, and worth owning in everyday travel. Prior research has mainly examined shared-use adoption, continuance, or direct effects of motives such as enjoyment, environmental concern, and economic benefit on intention [9–12]. Other studies have focused on city-level demand effects, operational viability, or governance [13–20]. What remains unclear is the mechanism through which value cues are translated into private purchase intention.

This study addresses that gap by integrating TAM/UTAUT, Self-Determination Theory (SDT), and the S-O-R framework. TAM/UTAUT provides the basis for positioning performance expectancy (PE) as the central appraisal in the model [7,8]. We therefore specify hedonic motivation (HM), future expectations (EF), and low price sensitivity (LPS) as antecedents of PE. SDT explains how PE is associated with purchase intention (PI) through perceived autonomy (PA) and perceived competence (PC), while innovative consumption (IC) captures consumers’ tendency to explore and experiment with new products. The study contributes in three ways. First, it shifts the focus from shared use to private ownership intention. Second, it explains how value cues operate through performance expectancy and then through motivational states rather than as isolated direct predictors. Third, it uses a sequential explanatory mixed-methods design to identify the practical conditions under which this mechanism is strengthened or weakened.

The remainder of the paper is organized as follows. We review the literature on micromobility adoption and develop the hypotheses. We then describe the data, measures, and modeling strategy, followed by the empirical results and the integration of qualitative evidence. We conclude with managerial implications for product strategy (battery and reliability features), pricing and segmentation (heterogeneous price sensitivity), and public-private governance (transit integration, parking, and safety standards), as well as limitations and directions for future research.

## 2. Study background

This section synthesizes recent peer-reviewed research on e-scooters and micromobility (2022–2025) through a management lens. We organize the evidence into four complementary strands: consumer motives and acceptance; city- and firm-level demand effects; operations and unit economics; and governance and equity. Table 1 summarizes representative studies across these strands to clarify what is already known, what remains underexplained, and where our study contributes.

**Table 1:** Synthesis of prior research on e-scooters and micromobility.

Study	Context & Sample	Method	Key Constructs	Main Findings / Managerial Takeaways
**[9]**	Turkey; 204 e-scooter users (emerging economy)	Survey; PLS-PM/SEM	Time-saving motivation; environmental awareness; continuance	Immediate/practical benefits dominate; environmental motives are secondary. In emerging markets, position offerings around time savings and convenience.
**[13]**	USA; 98 cities, 391 firms (city-level panel)	Quasi-experimental city entry; panel econometrics	Micromobility access; consumer spending	City e-scooter entry raises restaurant spending ~5.2%; boosts discovery and repeat visits. Clear demand-creation effects at the city/business level.
**[10]**	Turkey; 346 respondents	Survey; PLS-SEM (COVID moderator; IPMA)	Extended TAM: social influence, perceived pleasure, economic benefit → PU/PEOU → attitude/intention	High explanatory power; economic benefit increases use. Optimize price–value architecture to strengthen adoption.
**[17]**	Shared e-scooter service production (S3)	Operations economics model (swapping tours, DoD, BEC, lifespan/energy optimization)	Swappable batteries; cost function; inventory/charging logistics	Swap strategy drives total cost; closed-form optima for DoD/BEC/tours. Unit energy cost is far above grid price → logistics–operations integration is critical.
**[14]**	E-scooter sharing; news & user reviews	IS-AM (information-seeking argument mining)	Reasons for/against; argument salience; service design	Extracts 40 reasons with importance ranking; news provide richer arguments than reviews. Text mining is actionable for service improvement.
**[15]**	Louisville, KY; MDS data	Survival analysis (availability equity) + distributive equity model	Rebalancing equity; socio-demographics	Low-income/low-car areas see longer availability; minority-dominant areas face faster rebalancing, narrowing use windows. Embed equity frameworks in operations.
**[16]**	Palermo; city-scale socio-economic data	Machine learning (MLP best classifier)	Socio-economic factors; micromobility use	Middle-income and freelancers are more likely users. Not just a low-income/student mode → adjust segmentation and targeting.
**[11]**	Sweden; users (n = 337) + non-users (n = 1,001)	Survey; SEM	Eco-innovativeness; instrumental, environmental, hedonic, symbolic motives	Hedonic motivation is strongest; environmental motives negative for current use but positive for future intention. Messaging should go beyond “green”.
**[18]**	Five shared mobility types (incl. e-scooters)	Literature + expert interviews → cost modeling	Cost components; ops vs. leasing shares; viability	Operations + leasing >50% of cost/PKM. In e-scooters/bikes, field agents & maintenance dominate ops costs. Profitability is tenuous; utilization is the key lever.
**[12]**	Shared e-bikes & e-scooters; 800 users	Survey; SEM (four models)	Hedonic vs. environmental routes; DSI; green identity	E-bikes follow a cognitive route; e-scooters an affective/hedonic route. DSI → positive emotions; green identity affects environmental motives only for e-bikes.
**[19]**	US consumers; China & Cambodia products; categories include e-scooters vs. refrigerators	Discrete-choice conjoint	Mitigating weak COO: store image, warranty, third-party certification, corporate engagement	Retail store image is most effective; extended warranty second; certification third; corporate engagement least. Effects broadly robust across segments.
**[20]**	Hospitality industry; internal stakeholders & risks	Picture Fuzzy Sets–based MACTOR	Micromobility risks; stakeholders; sustainability	Maps risks (guest inexperience, helmet access, storage) and priority stakeholders. Managing these risks strengthens sustainability positioning and operations planning.

A first stream examines consumer motives and acceptance. Much of this work identifies hedonic, instrumental, and environmental motives as key predictors of intention or continuance, typically estimating relatively direct effects from motives to intention. For example, prior evidence highlights the prominence of immediate practical value and enjoyment in shaping acceptance, with environmental motives often playing a more nuanced or context-dependent role [9–12]. These studies provide strong descriptive insight into what correlates with adoption tendencies. However, they leave open a management-relevant question about mechanism: how do such motives become a willingness to commit to ownership, and through which psychological resources does that conversion occur?

A second stream shifts from individual motives to market outcomes, showing that micromobility can generate measurable demand and spillovers at the city and business level, and that user discourse can reveal actionable reasons for and against adoption. City-entry evidence suggests economically meaningful effects on local spending [13], while argument-mining approaches demonstrate how structured text analytics can surface prioritized service-improvement themes [14]. Related research also points to heterogeneity in who uses micromobility and in how availability is distributed across neighborhoods, highlighting demand segmentation and equity concerns [15,16]. This stream strengthens the case that micromobility matters operationally and commercially. However, it does not directly resolve the household-level purchase decision that anchors product strategy, after-sales design, and pricing architecture.

A third stream focuses on operations and unit economics, emphasizing that viability depends on logistics, maintenance, and the operating model design. Analytical work on battery swapping clarifies how reliability and cost structure are tightly coupled through operational decisions [17], and broader viability assessments indicate that field operations and maintenance can dominate cost, making utilization and service design central managerial levers [18]. This stream is highly relevant for managerial decision-making because it identifies the supply-side constraints that consumers ultimately experience, including reliability, convenience, and total cost of ownership. However, it is rarely connected explicitly to the psychological pathway through which consumers form performance-related beliefs and translate them into intention.Against this literature, this study advances purchase-intention research in three ways. It models HM, EF, and LPS as antecedents that shape private e-scooter purchase intention through PE and the motivational resources that make ownership feel actionable [9–12]. It aligns the dependent construct with private ownership decisions, including product reliability, safety, after-sales support, and pricing architecture [13–16]. It also adds a forward-looking belief component and treats price sensitivity as a value-evaluation channel, then uses interviews to identify contextual conditions that shape the mechanism, including infrastructure, safety, service access, warranty support, and total-cost transparency [17–20]. This approach explains how ownership intention is formed and why its strength depends on practical operating conditions.

## 3. Theoretical framework

**Fig 1:**
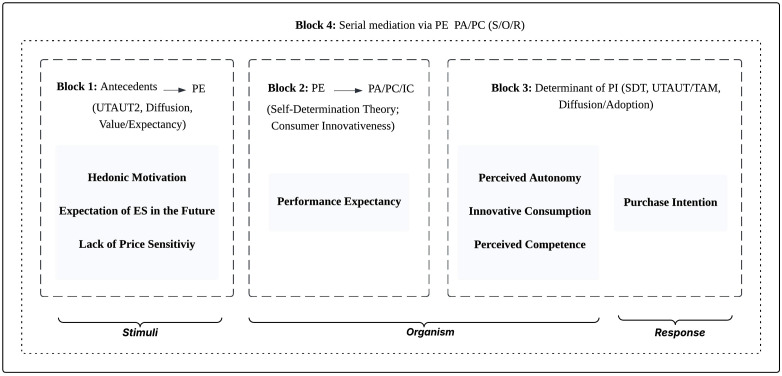
Theoretical scaffolding (S–O–R) behind the model.

We explain e-scooter PI through a process view that links external cues to internal appraisals and, ultimately, to commitment to ownership. We use Stimulus-Organism-Response (S-O-R) as an organizing scaffold: stimuli are evaluative cues present before a focal judgment is formed, the organism refers to the internal states that translate those cues into readiness to act, and the response captures the focal behavioral tendency [21]. This distinction is important for EF and PE. Although both involve expectation-related judgments, EF reflects a broad forward-looking evaluation of the technology’s trajectory, including diffusion, sustainability, and functional maturation, and is therefore treated as a stimulus. PE captures the focal appraisal of whether an e-scooter will improve personal travel performance through time savings, access, and reliability, and is therefore modeled as the central organismic appraisal. As shown in Fig 1, HM, EF, and LPS are treated as stimuli; PE is the central appraisal within the organism; PA, PC, and IC represent organismic states; and PI is the response. We draw selectively from UTAUT/UTAUT2 rather than reproducing the full model. PE is retained as the most proximal performance-related appraisal, HM as a salient experiential antecedent, and EF and LPS because private ownership decisions also depend on future viability and monetary sacrifice. Accordingly, HM, EF, and LPS are modeled as parallel antecedents of PE rather than as causal relations among themselves.

Within technology acceptance research, PE is consistently the appraisal most proximal to intention because it captures the consumer’s judgment that a technology will improve task performance [22]. For private e-scooter purchase, task performance is not an abstract concept; it reflects time savings, trip feasibility, reliability, and uninterrupted access for daily travel. We therefore place PE at the center of the mechanism and theorize that the main antecedents exert influence by shaping PE first, after which PE becomes consequential because it activates psychological resources that make ownership feel both workable and worthwhile.

HM is expected to raise PE because pleasurable, engaging experiences can heighten perceived task fit and usefulness. Technology adoption research links enjoyment to stronger usefulness beliefs through affect-to-cognition spillover and cognitive absorption [23,24]. In micromobility contexts, the same logic is visible: practical gains and enjoyment jointly contribute to favorable acceptance evaluations [9,10], and hedonic routes appear particularly salient for e-scooters relative to e-bikes [12]. Accordingly, we treat HM as a stimulus that strengthens PE by making benefits more vivid, easier to imagine, and more likely to be experienced as personally relevant.

EF captures forward-looking beliefs about whether the option will remain viable and continue to improve as technology and its ecosystem evolve. Expectancy and value theory emphasizes that anticipated outcomes shape utility appraisals and willingness to invest effort [25,26]. In our context, EF reflects expectations about sustainability relevance, diffusion, and availability, and functional maturation such as range reliability and perceived safety. Life-cycle evidence suggests that environmental performance improves with longer lifetimes and optimized logistics, which can rationalize more optimistic expectations and higher perceived usefulness [27]. Complementing this, city-level evidence showing spending spillovers after e-scooter entry is consistent with rising perceived usefulness of access and convenience [13]. Diffusion theory provides the macro-level link by explaining how such expectations influence perceived feasibility and adoption thresholds [28]. We therefore conceptualize EF as a stimulus that updates PE by shaping consumers’ beliefs about the option’s future task fit rather than merely its current attributes.

LPS is introduced to capture heterogeneity in how strongly consumers foreground monetary sacrifice when forming performance judgments. Value evaluation research conceptualizes perceived value as a comparison between expected benefits and perceived sacrifice [29]. When price sensitivity is low, the monetary sacrifice becomes less salient, and attention can shift toward functional benefits, raising perceived usefulness [30]. A closely related logic appears in acceptance research through price value, which links benefit perceptions relative to cost with adoption tendencies [31]. In micromobility, economic benefits and cost architecture are repeatedly highlighted as central to usage and viability [10,18], and operational accounts of battery swapping further clarify why reliability and range become focal when price concerns recede [17]. We therefore model LPS as a stimulus that elevates PE by reducing the weight of monetary sacrifice in evaluation and increasing the weight of functional performance considerations.

PE alone, however, does not fully explain why a favorable appraisal becomes a willingness to commit to ownership. We draw on Self-Determination Theory (SDT) to specify the psychological conversion mechanism that turns instrumental appraisals into volitional readiness. SDT argues that autonomy and competence are core needs that sustain self-endorsed choice and persistence [32]. When consumers believe an e-scooter will reliably deliver time savings and access, they should feel greater control over routing and timing, which supports PA, and greater confidence in using the mode effectively, which supports PC. Consistent with this reasoning, evidence in micromobility and allied service settings links accessibility and distribution features to autonomy-related experiences [15] and highlights the role of self-efficacy and competence for continued engagement [33,34]. In our framework, PA and PC are organismic states that transmit the effect of PE to PI because they explain why usefulness is experienced as personally actionable.

IC is treated as an approach-oriented organismic state rather than a terminal outcome. Innovativeness research suggests that trait and domain-specific innovativeness fosters exploratory adoption, experimentation, and deeper engagement with new products [35–37]. In micromobility, hedonic and innovative routes can be strong predictors of intention, especially for emerging green mobility innovations [11,12]. Building on this logic, we argue that when PE is high, exploration of features, new use cases, and personal routines becomes more attractive because the perceived payoff of experimentation increases and the perceived cost of trial decreases. IC, therefore, complements PA and PC by capturing an action orientation through which favorable appraisals can crystallize into purchase intention.The model yields a focused process logic. HM, EF, and LPS capture experiential value, future-oriented feasibility, and value evaluation. PE serves as the pivotal appraisal. PA, PC, and IC explain how PE is converted into ownership intention, drawing on SDT and innovativeness theory [32,35]. The empirical model tests whether HM, EF, and LPS influence PI through PE and then through PA, PC, or IC, as summarized in Fig 1 [21].

### 3.1. Hypotheses development

We expect HM, EF, and LPS to raise PE via affect-to-cognition spillovers, expectation updates regarding sustainability/diffusion/functional maturation, as well as benefit-over-sacrifice value appraisals. HM reliably heightens usefulness beliefs in consumer IT and in e‑scooter contexts [8,24,38,39]. EF should lift PE, consistent with lifecycle and city‑level evidence [13,27,40]. Low price salience shifts attention from monetary sacrifice to benefits, strengthening instrumental appraisals like PE [8,29,30,41].

**H1a.** HM is positively associated with PE.
**H1b.** EF of e-scooter technology are positively and significantly associated with PE.
**H1c.** LPS is positively and significantly associated with PE.

Consistent with SDT and post-adoption IS models, PE, the proximal appraisal of task-fit (time, access, reliability), is expected to translate into autonomy, exploration, purchase intention, and competence. Higher PE lifts PA in trip planning [42], catalyzes IC through lower experimentation costs and competence signals [43,44], predicts PI in micromobility models [45,46], and nurtures PC when tools are appraised as effective and autonomy-supportive [47].

**H2a.** PE is positively associated with PA.
**H2b.** PE is positively associated with IC.
**H2c.** PE is positively associated with PI.
**H2d.** PE is positively associated with PC.

Grounded in SDT, PA and PC operate as proximal motivational resources that translate functional appraisals into PI, while IC captures consumers’ propensity to trial and champion novel mobility solutions. Cross‑domain evidence shows that satisfying autonomy and competence needs significantly lifts PI in digital commerce [48]. In the transport domain specifically, an EV purchase model demonstrates that PA and PC foster IC, and, critically, IC strongly predicts EV purchase intention (β ≈ 0.55), establishing a clear motivational route from PA/PC to PI via IC [49]. Within micromobility, consumer innovativeness is a robust driver of use decisions and future behavioral intentions for shared e‑scooters and e‑bikes, underscoring the PI‑relevance of IC in this category [11]. Competence‑proximal beliefs also matter for mobility adoption: travel‑related self‑efficacy (a competence analogue) significantly increases intention to keep using community‑based electric ride‑sharing, aligning with a positive PC → PI pathway [34].

**H3a.** PA is positively associated with PI.
**H3b.** IC is positively associated with PI.
**H3c.** PC is positively associated with PI.

Accordingly, we specify serial paths in which HM/EF/LPS elevate PE, which then satisfies psychological levers culminating in PI. Enjoyable use heightens PE [8,23,24]. In micromobility, higher PE signals tangible functional gains (range, time, reliability) that expand autonomy and strengthen competence; PE also catalyzes innovative consumption by lowering experimentation costs and signaling competence [34,50,51]. In turn, autonomy, competence, and innovative consumption translate into higher PI, consistent with evidence that value/acceptance variables, including PE and enjoyment, enhance intentions and continuance [49,52].

**H4a.** HM positively influences PI through the sequential mediating effects of PE and PA.
**H4b.** HM positively influences PI through the sequential mediating effects of PE and PC.
**H4c.** HM positively influences PI through the sequential mediating effects of PE and IC.

Users’ EF forward-looking assessments of environmental impact, diffusion, and technological maturity update instrumental beliefs, most notably PE. Environmental value propositions and functional gains predict adoption and loyalty [40]. Recent LCAs show that improvements in durability, range, and logistics materially reduce e-scooters’ carbon footprint, rationalizing more optimistic expectations and reinforcing PE [27]. City-scale surveys likewise portray e-scooters as sustainable, efficient last-mile options, with comfort/ease-of-use facets of PE shaping intentions [53]. Higher PE enhances PA via perceived accessibility [54], strengthens PC through travel self-efficacy [34], and catalyzes IC as users explore advanced features [55]. Consistent with TAM/UTAUT and e-scooter evidence, PE, together with service-quality components, predicts purchase and continuance; the PE–loyalty–intention link is pronounced in multimodal contexts (e-scooter–metro), where perceived service quality and system acceptance drive sustained use [52]. Collectively, these findings support our sequential path model.

**H5a.** EF positively influence PI through the sequential mediating effects of PE and PA.
**H5b.** EF positively influence PI through the sequential mediating effects of PE and PC.
**H5c.** EF positively influence PI through the sequential mediating effects of PE and IC.

LPS down-weights monetary sacrifice and shifts attention to benefits/functionality; under benefit-focused appraisals, value/quality expectations rise and price salience no longer depresses benefit-based judgments, strengthening PE [56–58]. In micromobility, heterogeneous price sensitivity and perceptions of affordability/fairness shape preferences and downstream outcomes (e.g., satisfaction/loyalty) [59,60]. Once PE is high, users report greater autonomy via perceived accessibility and control, higher competence through reduced performance risk and self-efficacy, and more innovative exploration [11,34,54,61]. Consistent with e-scooter evidence, PE and service-quality facets also explain purchase and continuance intentions, anchoring the final link to PI [52,62]. Accordingly, we specify three serial paths:

**H6a.** LPS positively influences PI through the sequential mediating effects of PE and PA.
**H6b.** LPS positively influences PI through the sequential mediating effects of PE and PC.
**H6c.** LPS positively influences PI through the sequential mediating effects of PE and IC.

**Fig 2:**
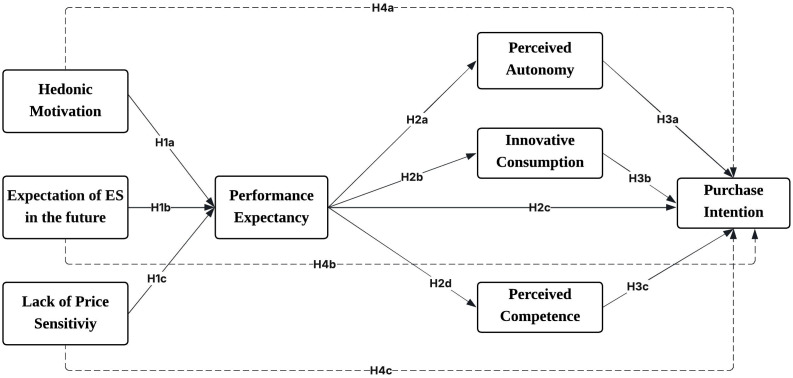
Research model.

Building on the above theoretical and empirical evidence, we propose the conceptual research model in Fig 2.

## 4. Methodology

### 4.1. Phase I: Quantitative strand

We employed partial least squares structural equation modeling (PLS-SEM) in SmartPLS 4, as the study is prediction-oriented and specifies a complex model with multiple endogenous constructs and serial mediations. Prior to estimation, the data were screened for missing values, response quality, and multicollinearity. Path significance was evaluated using 5,000 bias-corrected and accelerated (BCa) bootstrap resamples and two-tailed 95% confidence intervals. Out-of-sample predictive performance was assessed using PLSpredict with 10-fold cross-validation repeated 10 times [63,64].

#### 4.1.1. Data collection and sampling.

The target population for the quantitative strand comprised adults residing in Turkey who were aware of e-scooters and could evaluate private e-scooter ownership as a personal mobility option. Because the study examines purchase intention at the pre-purchase stage, prior ownership or regular use was not required. Respondents were recruited through an online voluntary participation call using convenience sampling. To be included, participants had to be at least 18 years old, reside in Turkey, provide informed consent, and complete the questionnaire. After screening for completeness and response quality, 338 valid responses were retained for analysis [64]. This sampling frame is appropriate because the study focuses on how consumers form ownership intention before purchase rather than on post-purchase behavior. Participant demographics are presented in Table 2. The study protocol was approved by the Yıldız Technical University Social and Human Sciences Research Ethics Committee (02 January 2025; Meeting No: 2025.01; Report No: 20250104092). Recruitment for the online survey took place from 10 January 2025–08 March 2025. All participants were adults (≥18 years). Prior to participation, all survey respondents were presented with a participant information sheet and provided informed consent electronically before accessing the questionnaire (i.e., consent was indicated by actively agreeing and proceeding to the survey). Written consent for face-to-face interviews and recorded verbal consent for online interviews; consent included permission to audio-record. No minors were included; therefore, parental/guardian consent was not applicable. The requirement for informed consent was not waived by the ethics committee.

**Table 2:** Participant demographics.

Variables	Categories	N	(%)
**Gender**	Female Male	161 177	47.6 52.4
**Age**	18-25	256	75.7
	26-35	68	20.1
	36-45	13	3.8
	46-55	1	0.3
**Education**	High school or below	14	4.1
	Vocational school	6	1.8
	Bachelor’s degree	281	83.1
	Master’s/PhD degree	37	10.9
**Occupation**	Student	252	74.6
	Homemaker	5	1.5
	Private sector employee	40	11.8
	Public sector employee	35	10.4
	Small business owner / Entrepreneur	4	1.2
	Unemployed / Not working	2	0.6
	Retired	13	3.8
**Average Income**	−22.104	10	3.0
	22.105-35.000	26	7.7
	35.001-55.000	26	10.7
	55.001-85.000	73	21.6
	85.001-120.000	76	22.5
	120.001-	117	34.6

n: 338 sample size.

All items were measured on a five-point Likert scale (1 = Strongly disagree, 5 = Strongly agree). The questionnaire was pretested to assess wording clarity and completion flow, and the average completion time was six minutes. Constructs were adapted from validated multi-item scales, and the exact item wordings with sources are reported in Supplementary Table S1 in S1 File. All constructs, including EF and LPS, were modeled as reflective latent constructs. This specification is consistent with the measurement design, in which the indicators were treated as manifestations of the underlying latent evaluations and assessed using internal consistency, convergent validity, and discriminant validity criteria.

Because the quantitative strand relied on self-reported survey data, procedural and statistical measures were employed to address common method bias. Respondents were assured of anonymity, predictor and criterion constructs were separated across questionnaire sections, and item order was mixed where appropriate. Full collinearity VIF values ranged from 1.284 to 2.417, remaining below the conservative threshold of 3.3 and indicating that common method bias was unlikely to materially affect the results. Detailed VIF values are provided in Supplementary Table S2 in S1 File.

#### 4.1.2. Measures and instrument development.

The measurement model demonstrated satisfactory reliability and validity across all constructs. As shown in Table 3, Cronbach’s alpha (.738–.892) and composite reliability (.847–.933) values were well above the recommended threshold of .70 [65], while AVE values ranged from .529 to .822, surpassing the .50 benchmark and thereby confirming convergent validity [66]. Moreover, the square root of AVE for each construct exceeded its correlations with other constructs, indicating adequate discriminant validity under the Fornell–Larcker criterion. For additional robustness, the HTMT ratios were examined (Table 4), all of which fell between .184 and .759, below the conservative cut-off value of .85 [67]. These results confirm the adequacy of the measurement model.

**Table 3:** Fornell-Larcker criterion.

	Cronbach’s alpha	CR	AVE	EF	HM	IC	LPS	PA	PC	PE	PI
**EF**	.892	.933	.822	**.907**							
**HM**	.738	.851	.659	.387	**.812**						
**IC**	.832	.873	.580	.358	.527	**.762**					
**LPS**	.835	.875	.584	.350	.621	.435	**.764**				
**PA**	.790	.847	.529	.179	.303	.327	.422	**.727**			
**PC**	.814	.871	.576	.316	.452	.615	.299	.408	**.759**		
**PE**	.777	.854	.595	.491	.481	.480	.315	.486	.489	**.771**	
**PI**	.876	.915	.731	.412	.631	.472	.330	.614	.523	.492	**.855**

**Table 4:** Heterotrait-Monotrait (HTMT) criterion.

	EF	HM	IC	LPS	PA	PC	PE	PI
**EF**								
**HM**	.428							
**IC**	.357	.715						
**LPS**	.284	.325	.345					
**PA**	.353	.220	.330	.349				
**PC**	.256	.426	.418	.337	.606			
**PE**	.237	.437	.300	.343	.459	.609		
**PI**	.424	.412	.405	.312	.307	.304	.478	

Discriminant validity was examined via cross-loadings (Table 5). Cross-loadings indicated that every indicator loaded highest on its intended construct and lower on all non-target constructs, supporting discriminant validity [68]. Item loadings and descriptive statistics, which report standardized loadings together with item means and standard deviations; all loadings met the .70 benchmark, and multicollinearity was assessed using VIFs (see Table S3 in S1 File). VIF Diagnostics; all values were below the conservative threshold of 3.3 (max = 2.98), indicating no problematic collinearity [64].

**Table 5:** Loadings and cross-loadings.

	EF	HM	IC	LPS	PA	PC	PE	PI
EF1	.828	.560	.400	.289	.318	.544	.417	.480
EF2	.874	.440	.418	.277	.272	.559	.404	.477
EF3	.928	.477	.415	.312	.358	.584	.479	.407
HM1	.445	.800	.322	.195	.248	.720	.407	.448
HM2	.617	.908	.370	.315	.261	.342	.451	.606
HM3	.416	.717	.331	.201	.270	.503	.314	.433
IC1	.214	.269	.706	.378	.202	.309	.214	.235
IC2	.236	.307	.710	.447	.198	.333	.168	.239
IC3	.245	.246	.797	.396	.291	.318	.221	.263
IC4	.272	.292	.796	.399	.292	.354	.249	.281
IC5	.247	.207	.793	.124	.100	.221	.229	.255
LPS1	.292	.265	.453	.752	.258	.286	.322	.314
LPS2	.217	.194	.374	.801	.035	.204	.160	.219
LPS3	.295	.274	.435	.857	.175	.272	.298	.297
LPS4	.139	.166	.337	.699	−.011	.140	.101	.119
LPS5	.213	.186	.409	.715	−.045	.167	.175	.197
PA1	.206	.193	.333	.139	.752	.253	.312	.252
PA2	.284	.254	.268	.075	.832	.271	.385	.322
PA3	.127	.151	.106	−.010	.836	.207	.251	.175
PA4	.104	.136	.183	−.022	.781	.171	.147	.135
PA5	.387	.121	.164	−.075	.764	.332	.509	.427
PC1	.445	.300	.322	.105	.248	.720	.207	.248
PC2	.617	.708	.370	.315	.261	.842	.451	.406
PC3	.416	.316	.331	.201	.270	.773	.314	.433
PC4	.458	.470	.407	.225	.266	.806	.319	.482
PC5	.371	.456	.359	.173	.333	.709	.305	.387
PE1	.512	.521	.356	.246	.385	.523	.827	.537
PE2	.298	.284	.264	.149	.454	.287	.769	.351
PE3	.271	.264	.287	.251	.324	.246	.732	.298
PE4	.336	.365	.238	.310	.360	.358	.756	.355
PI1	.657	.560	.400	.289	.318	.544	.417	.874
PI2	.543	.540	.418	.277	.272	.559	.404	.871
PI3	.723	.577	.415	.312	.358	.584	.479	.907
PI4	.501	.428	.367	.234	.458	.467	.482	.764

### 4.2. Phase II: Qualitative strand

Phase II thematically analyzes 26 semi-structured interviews to contextualize and extend the PLS-SEM findings. Using a hybrid deductive–inductive protocol anchored in PE, PA, PC, IC, EF, LPS, and HM, the research team iteratively coded transcripts and maintained an audit trail; thematic sufficiency was reached.

#### 4.2.1. Sampling and data collection.

For the qualitative strand, we used criterion-based purposive sampling of survey respondents who were (i) ≥18, (ii) urban residents in Turkey, (iii) aware of e-scooters and either recent users or non-users with purchase intention, and (iv) consented to re-contact. To broaden heterogeneity, we applied maximum-variation sampling across usage status (current, former, potential), gender, age, income, education, urban context, commuting patterns, and tertiles of latent scores (PE, HM, EF, PA, PC, IC, LPS). We conducted 26 semi-structured interviews (45–60 minutes), either online or face-to-face, according to the participant’s preference.

The study protocol was approved by the Yıldız Technical University Social and Human Sciences Research Ethics Committee (02 January 2025). Recruitment for the semi-structured interviews took place from 30 May 2025–12 July 2025. All interview participants were adults (≥18 years). Prior to the interview, participants received a participant information sheet and provided informed consent. For face-to-face interviews, consent was obtained in writing. For online interviews, consent was obtained verbally and was audio-recorded at the beginning of the session and documented by the interviewer; consent also included permission to audio-record the interview. No minors were included; therefore, parental/guardian consent was not applicable. The requirement for informed consent was not waived by the ethics committee.

The sample size was set a priori using the information-power principle: given a focused aim, a theory-informed guide (UTAUT2, SDT), and a bounded frame, 20–30 cases were deemed adequate. We monitored saturation during fieldwork, and code saturation emerged around interview 19, indicating saturation by interview 24. Two additional cases (25–26) were included to test thematic stability and capture edge profiles (e.g., high innovativeness yet risk-averse).

Recruitment relied on re-contacting permitted survey respondents, as well as outreach to mailing lists, online communities, and mobility networks. A brief screener verified eligibility and quotas. All interviews were obtained with consent, recorded verbatim, transcribed, de-identified, and managed in accordance with approved ethics and confidentiality protocols. Table 6 summarizes participant characteristics. Detailed participant information (e.g., interview duration, commute time, infrastructure access, scooter experience) is provided in Table S4 in S1 File**.**

**Table 6:** Sample characteristics of qualitative participants.

ID	Gender	Age	Education	Occupation	Income * ₺
**P01**	Female	18-25	Bachelor’s	Student	22.501–39.999
**P02**	Male	26-35	Bachelor’s	Sales professional	40.000–59.999
**P03**	Female	26-35	Master’s	Academic professional	22.501–39.999
**P04**	Male	26-35	Bachelor’s	Technology professional	≥ 80.000
**P05**	Female	26-35	Bachelor’s	Design professional	40.000–59.999
**P06**	Male	18-25	High School	Transport worker	22.501–39.999
**P07**	Female	36-45	Master’s	Education professional	60.000–79.999
**P08**	Male	26-35	Bachelor’s	Engineering professional	60.000–79.999
**P09**	Male	26-35	Bachelor’s	Marketing professional	40.000–59.999
**P10**	Male	26-35	Master’s	Managerial professional	≥ 80.000
**P11**	Male	36-45	Bachelor’s	Managerial professional	≥ 80.000
**P12**	Female	18-25	Associate	Service employee	22.501–39.999
**P13**	Male	26-35	PhD	Academic professional	60.000–79.999
**P14**	Female	36-45	Bachelor’s	Healthcare professional	40.000–59.999
**P15**	Male	36-45	High School	Transport worker	40.000–59.999
**P16**	Female	18-25	Bachelor’s	Student	≤ 22.500
**P17**	Male	26-35	Master’s	Data professional	60.000–79.999
**P18**	Female	26-35	Bachelor’s	Human resources professional	60.000–79.999
**P19**	Male	18-25	Bachelor’s	Design professional	22.501–39.999
**P20**	Female	26-35	Master’s	Legal professional	≥ 80.000
**P21**	Male	26-35	Bachelor’s	Business owner	≥ 80.000
**P22**	Male	36-45	Bachelor’s	Administrative employee	40.000–59.999
**P23**	Male	26-35	Associate	Technical worker	40.000–59.999
**P24**	Female	26-35	Master’s	Finance professional	60.000–79.999
**P25**	Male	36-45	PhD	Academic professional	≥ 80.000
**P26**	Female	36-45	Bachelor’s	Business owner	≥ 80.000

* ₺ denotes Turkish lira (TRY).

#### 4.2.2. Data collection procedures and analysis.

All interviews were de-identified, transcribed verbatim in Turkish, and managed in MAXQDA. We conducted reflexive thematic analysis following Braun and Clarke [69,70]. Our coding strategy was hybrid: (i) deductive codes derived from the conceptual model (PE; HM; EF; LPS; PA; PC; IC; PI pathways), and (ii) inductive codes capturing unanticipated mechanisms and boundary conditions (e.g., infrastructure & safety constraints, perceived regulatory trust, service/repair availability, social norms, environmental identity). The full semi-structured interview guide, including main questions and probes, is provided as S5 in S1 File.

Two trained researchers double-coded an initial calibration subset (6 transcripts; ~ 23%) to develop and stabilize the codebook. During this phase, Cohen’s κ for principal codes ranged .81–.90 (M = .86) [71]. We report κ only for calibration purposes to document shared understanding; subsequent analysis followed reflexive TA principles. Discrepancies were resolved by discussion and codebook refinement. One researcher then coded the full corpus, with scheduled peer debriefs to examine interpretations, track negative cases, and maintain analytic consistency. We kept an audit trail (decision logs, code changes) and reflexive memos throughout. Because the study was theory-informed, the interviewers remained attentive to the risk of imposing prior expectations on the data. Interviews followed a shared semi-structured guide, and probing was used to clarify participants’ meanings. Interpretive bias was managed through calibration coding, peer debriefing, an audit trail, and reflexive memoing.

The interview guide was informed by the quantitative findings and the conceptual model. All interviews were audio-recorded with consent, transcribed verbatim, and coded in iterative rounds. The analysis moved from theory-driven initial codes to inductively refined themes through memoing, peer discussion, and repeated comparison across cases. The 26 interviews yielded ~80,700 words of transcribed text. Individual transcripts ranged from ~2,500 to ~4,000 words (M ≈ 3,105; SD ≈ 430). This corpus provided sufficient information power and meaning saturation for theme development: code saturation was reached by interview 19, and meaning saturation by 24; the final two interviews were retained to test thematic stability and probe edge cases (e.g., high-innovation interest but strong safety aversion).

## 5. Result

### 5.1. Quantitative results

**Fig 3:**
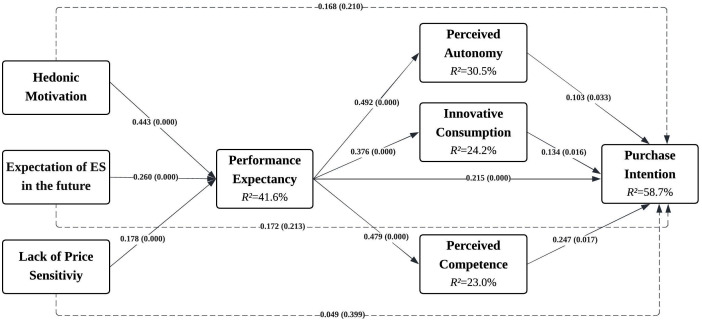
Research model structural path diagram.

We report the structural model estimates from the PLS-SEM analysis. Fig 3 displays standardized path coefficients; significance is evaluated with 5,000-sample BCa bootstrapping (two-tailed 95% CIs), and indirect/serial effects are judged by the non-inclusion of zero. The original, full-resolution PLS path diagram is provided as S1 Fig.

We summarize explanatory power (R²), effect sizes (f²), and out-of-sample predictive performance using PLSpredict with k-fold cross-validation; multicollinearity is not a concern (all VIF < 3.3; see Table S6 in S1 File). The accompanying tables list R²/Q²_predict values and the complete set of direct and mediated paths (H1–H6) that guide the interpretation that follows.

The PLSpredict results yielded positive Q²_predict values for all endogenous constructs (e.g., PI = .568; PE = .409), demonstrating that the model exceeds the naïve (IA) benchmark and provides out-of-sample predictive validity [63]. Compared to the linear model (LM), PLS produced lower RMSE and MAE values for PA, PE, and PI, whereas PC and IC exhibited marginally higher error levels in favor of LM (Table 7). The R² values further indicated moderate explanatory power for PI (.587), weak-to-moderate levels for PE (.416) and PA (.305), and weak explanatory power for IC (.242) and PC (.230) [64]. When confirmed at the indicator level, the overall predictive performance of the model can be considered “moderate.” Nevertheless, the relatively weaker prediction for PC and IC may be improved by considering potential nonlinear effects, interaction terms, or unobserved heterogeneity through advanced procedures such as RESET tests, PLS interaction modeling, or segmentation techniques like FIMIX or PLS-POS [68].

**Table 7:** Out-of-sample predictive performance.

Construct	R²	Q²_predict	RMSE P|LM (Δ)	MAE P|LM (Δ)	Verdict
**PA**	0.305	.298	.824 | .842 (−.018)	.713 | .731 (−.018)	Better
**PC**	0.230	.222	.791 | .780 (+ .011)	.625 | .617 (+.008)	Worse
**PE**	0.416	.409	.749 | .758 (−.009)	.552 | .560 (−.008)	Better
**PI**	0.587	.568	.776 | .790 (−.014)	.626 | .638 (−.012)	Better
**IC**	0.242	.239	.808 | .800 (+ .008)	.704 | .695 (+.009)	Worse

Note: PLSpredict was conducted using 10-fold cross-validation with 10 repetitions and the fixed-seed option enabled. Q²_predict > 0 indicates predictive performance beyond the indicator-average benchmark. Δ = PLS − LM; negative values favor PLS.

All proposed direct effects were examined (Tables 8 and 9). H1a was supported (β = .443, p < .001; *f*² = .201, medium), indicating that HM positively predicts PE. H1b (β = .260, p < .001; *f*² = .178, medium) and H1c (β = .178, p < .001; *f*² = .097, small) were also supported, suggesting that EF meaningfully and LPS modestly enhance PE. Regarding downstream effects, H2a was supported (β = .492, p < .001; *f*² = .318, medium–large) and H2d was supported (β = .479, p < .001; *f*² = .301, medium–large), showing that PE most strongly shifts PA and PC. H2b was supported (β = .376, p < .001; *f*² = .165, medium), while H2c, the direct link to intention, was smaller yet significant (β = .215, p < .001; *f*² = .109, small), consistent with PE’s influence on PI being transmitted largely through the psychological mechanisms. For the outcome stage, H3a (β = .103, p = .033; *f*² = .115, small) and H3b (β = .134, p = .016; *f*² = .092, small) were supported, whereas H3c emerged as the most consequential outcome path (β = .247, p < .001; *f*² = .233, medium), implying that competence contributes more strongly to intention than autonomy or innovativeness.

**Table 8:** Results of hypothesis testing.

Hyp.	β	SD	t-value	95% CI (2.5%–97.5%)	p-value	f²
**H1a.**	.443	.041	10.773	[.328, .558]	.000***	**.201**
**H1b.**	.260	.058	4.442	[.149, .371]	.000***	**.178**
**H1c.**	.178	.051	3.521	[.090, .266]	.000***	**.097**
**H2a.**	.492	.041	12.064	[.412, .572]	.000***	**.318**
**H2b.**	.376	.047	7.992	[.283, .469]	.000***	**.165**
**H2c.**	.215	.046	4.690	[.125, .305]	.000***	**.109**
**H2d.**	.479	.042	11.461	[.396, .562]	.000***	**.301**
**H3a.**	.103	.044	2.325	[.007, .199]	.033*	**.115**
**H3b.**	.134	.041	3.250	[.042, .226]	.016*	**.092**
**H3c.**	.247	.031	8.098	[.145, .349]	.000***	**.233**
**H4a.**	.113	.031	3.636	[.038, .188]	.001**	–
**H4b.**	.098	.030	3.246	[.033, .163]	.001**	–
**H4c.**	.000	.002	0.016	[-.004, .004]	.987 (ns)	–
**H5a.**	.111	.040	2.810	[.032, .190]	.004**	–
**H5b.**	.152	.037	4.066	[.079, .225]	.000***	–
**H5c.**	.000	.001	0.016	[-.003, .003]	.987 (ns)	–
**H6a.**	.127	.035	3.673	[.051, .203]	.000***	–
**H6b.**	.101	.034	2.976	[.032, .170]	.006**	–
**H6c.**	.000	.001	.015	[-.003, .003]	.988 (ns)	–

Note: “Supported” = two-tailed 95% bootstrap CI excludes zero. Significance: *p* < .05*, p < .01**, p < .001***, ns. = p ≥ 0.05, *f*² (direct paths): .02/.15/.35 [72].

**Table 9:** Mediation diagnostics: direct and indirect effects (BCa bootstrap).

Path	β	SD	T-value	P-value
**LPS → PI**	.049	.057	.824	.399
**HM → PI**	.168	.133	1.323	.210
**EF → PI**	,172	,128	1,345	,213

Turning to the serial-mediation hypotheses, H4a (HM → PE → PA → PI; β = .113, p = .001) and H4b (β = .098, p = .001) were supported, while H4c was not (β ≈ .000, p = .987). The same pattern held for EF (H5a supported; H5b supported; H5c not supported) and for LPS (H6a and H6b supported; H6c not supported). The IC-related results require a more precise interpretation. PE → IC showed a medium effect size (*f*² = .165), whereas IC → PI showed a small effect size (*f*² = .092). The unsupported serial paths through IC indicate that innovativeness did not transmit the upstream effects of HM, EF, and LPS to PI in this model, although IC had a small direct association with PI.. Finally, the complementary direct-effect tests (Table 9) showed that HM, EF, and LPS do not directly predict PI (p = .210, .213, .399), consistent with indirect-only mediation via PE→(PA/PC). The *f*² profile (≈ .09–.33; small ≈ .02, medium ≈ .15, large ≈ .35) underscores a mechanism in which antecedents raise PE (moderate effects), which in turn meaningfully boosts autonomy and competence (medium–large), thereby elevating PI [72]. These indirect effects should be interpreted as consistent with the proposed process model rather than as evidence of temporal causality, because the quantitative strand is cross-sectional and the ordering among PE, PA, PC, and PI is theory-driven rather than directly observed.

### 5.2. Qualitative results

We integrated strands via matrix queries and joint displays that align themes/codes with the PLS-SEM paths. Quotations were purposively sampled for heterogeneity (usage status, gender, age, city size), translated by a bilingual researcher, and back-checked. The final scheme comprises six themes and sixteen sub-themes; Table S7 in S1 File reports definitions, counts, and links to quantitative paths. In brief, the themes corroborate the serial pathway PE elevates PA/PC and fosters IC while price salience and future expectations condition this conversion. We presented each theme with a concise definition, one or two quotations, and its connection to the relevant path(s).

#### 5.2.1. Theme 1: Time as the currency: Function‑first adoption.

Across most interviews (21/26), participants framed e‑scooters foremost as time‑saving, door‑to‑door mobility tools. Pleasure and novelty were acknowledged, yet they rarely anchored the decision; instead, respondents evaluated scooters through a strict utility calculus: Does it shorten my routine trips, reduce waiting, and make arrival times predictable? This function‑first stance clarifies why PE sits at the center of the mechanism and why its effect on intention is transmitted primarily via autonomy and competence. Saving time enables self‑set schedules (autonomy) and routinized mastery (competence), which together make purchase more likely.

Participants repeatedly described the last 2–3 kilometers after metro or tram as the critical bottleneck. When the scooter reliably compresses the “last mile” and removes the need to wait for feeder buses, perceived control over timing increases and commuting becomes more consistent day‑to‑day. As one current user put it:

“*From the metro to my office it’s about two to three kilometers. With the scooter I stop waiting for a bus, door‑to‑door becomes predictable.*” (P10)

Utility also hinged on range adequacy and ride comfort (single‑charge coverage; handling uneven surfaces). Where these held, scooters were folded into multimodal routines and daily schedules were retimed—consistent with PE → PA → PI.

Counter‑narratives stressed contextual limits: poor surfaces, steep slopes, or aggressive traffic eroded the time advantage:

“*On my route drivers don’t respect lanes, and the pavement is broken. Some days the scooter is fast; other days I lose time dodging risks. If it can’t be consistent, I don’t see the point of buying.*” (P26)

Theme 1 shows that purchase intention is catalyzed when scooters consistently convert distance into time saved in everyday conditions. The hedonic upside was welcomed but secondary; participants were swayed when the device made commutes predictably shorter, thereby raising autonomy (self‑set timing) and consolidating competence (routine mastery). Where infrastructure and traffic norms undercut predictability, the same participants withdrew or deferred purchase, underscoring that **time predictability—not raw speed—**is the decisive currency in adoption.

#### 5.2.2. Theme 2: The autonomy dividend: Self‑set schedules and door‑proximity parking.

Participants consistently described e‑scooters as a way to decouple daily movement from external timetables (buses, ride‑hailing, carpools) and to park within meters of the destination. Autonomy here was practical rather than abstract: leaving exactly when needed, rerouting on the fly, and locking the scooter at the door. This pattern explains why PE translated into PA and, in turn, into purchase intention, time saved becomes time controlled. Where rules and parking zones were clear, autonomy felt effortless; where signage or enforcement was inconsistent, perceived freedom turned into friction.

“*On busy days I can’t wait for a car or the feeder bus. With the scooter I leave when I decide, and I lock it right by the entrance. That control over timing is what makes it viable*.” (P20)

“*In this city center is tricky, parking rules aren’t always clear. I loved skipping the wait, but the risk of a fine or conflict ate into that freedom. In the end I went back to the bus.*” (P22)

Autonomy is the active conduit: when riders can self‑set schedules and park at the door, intention rises (PE → PA → PI). Regulatory clarity and parking supply are boundary conditions that either amplify or blunt the autonomy dividend.

#### 5.2.3. Theme 3: The competence ramp: From hesitation to routine mastery.

A second mechanism was a short learning curve that moved participants from cautious trial to confident, routinized riding. Riders described acquiring micro‑skills, braking modulation, lane positioning, anticipating drivers/pedestrians, and adopting safety routines (helmet, speed discipline, route timing). Once this competence “clicked,” purchase started to feel justified, aligning with the strong PE → PC and PC → PI links observed quantitatively. However, competence was context‑sensitive: hostile traffic or poor surfaces could reset confidence, even after weeks of practice.

“*The first three days I rode like I was holding on for dear life. Then I learned to read the gaps and modulate brakes. That was the moment I thought, ‘okay, buying makes sense now.*’” (P17)

“*I practiced a lot and felt fine, but one close call with a minibus shook me. My skills were there, yet the infrastructure wasn’t; I sold the scooter after that.*” (P11)

Interpretation. Competence is the proximal driver of intention: as self‑efficacy consolidates, purchase becomes rational and safe in the rider’s mind (PE → PC → PI). Still, the competence ramp depends on context, which lane protection and surface quality can either reinforce or erode newly formed confidence.

#### 5.2.4. Theme 4: Price as threshold: Value calculus over novelty.

This theme surfaced in 25 of 26 interviews. Participants did not treat price as a lever that *pulls* intention upward; rather, price set a threshold that either removes friction when value is clear or blocks adoption when it is not. The dominant calculus was pragmatic: *If the scooter reliably saves time and fits my routes, I’ll pay; if not, even cheap is expensive.* In practice, willingness‑to‑pay rose for performance‑relevant features (range, braking, suspension) that protect the time advantage, whereas cosmetic or novelty features carried little weight. Many participants described back‑of‑the‑envelope payback thinking (minutes saved × commuting days), but only after the device had proven itself in their actual conditions.

“*If it cuts thirty minutes a day and doesn’t fail on my route, the higher trim pays for itself. But if service is spotty and I lose time when it’s in repair, the math collapses*.” (P21)

Beyond purchase price, respondents weighed the total cost of ownership (TCO) battery longevity, parts/service access, depreciation, and theft/insurance risk. For frequent riders, warranty and replaceable‑battery options were framed as “time insurance”: paying more upfront to avoid downtime later. For lighter or seasonal use, rental/subscription often outcompeted ownership because it offloaded TCO and theft anxiety, even if the per‑ride price was higher.

“*I stretched for the model with better brakes and a replaceable battery; the extra cost felt like paying for reliability. My logic wasn’t fancy, just fewer delays, fewer headaches, and I keep using it through the year*.” (P25)

Counter‑views cautioned that uncertain TCO, especially battery replacement and winter usage, can flip the equation. Several participants preferred to keep renting until usage stabilized; others highlighted that promotions mattered only after baseline utility was met. An unfinished thought, voiced by more than one interviewee, captures this risk calculus:

*“…if the battery needs replacing in year two and the warranty doesn’t cover it, then…”* (P18)

Price rarely generates intention on its own; it functions as a value gate. When performance is credible, and the ecosystem (service, parts, insurance) limits hidden costs, participants cross the gate and accept higher upfront prices consistent with LPS influencing intention indirectly via performance expectancy and, downstream, autonomy/competence (LPS → PE → PA/PC → PI). When TCO is opaque or context erodes performance (poor surfaces, theft risk, seasonal gaps), the same participants defer purchase or remain with rental, aligning with the non‑significant direct LPS → PI path in the structural model.

#### 5.2.5. Theme 5: Innovation ≠ Identity: Utility before novelty.

This theme was evident in 24 of 26 interviews. Participants generally enjoyed the novelty and “tech” appeal of e‑scooters, yet they rarely treated innovation as a decisive reason to buy. The “cool factor” operated as a secondary amplifier useful only after core utility (time saved, reliable range, acceptable ride comfort) was demonstrated. Many also expressed image ambivalence: they did not want to be labeled “the scooter person,” and several noted that novelty can attract unwanted attention or social judgment in dense urban settings. This helps explain why IC showed at best a small direct link to intention and did not carry the antecedents through serial mediation; in practice, function and control outweighed novelty.

“I like trying new devices, but I won’t buy to look like an early adopter. If it doesn’t change my day, it’s just an expensive toy.” (P09)

“At work I became the ‘scooter guy’…..fun at first, but the novelty wore off and the lanes weren’t safe on my route. I like new tech, yet function didn’t hold, so I sold it.” (P11)

A minority counter‑view acknowledged that innovation can spark trial, but even here, retention hinged on proven utility:

“*The first‑of‑its‑kind feeling made me test it. I only kept it when I could cut 20–25 minutes on a normal day. Without that, I’d have returned it.*” (P25)

IC functions as a curiosity trigger rather than a purchase engine. It can open the door to trial (and indirectly help people learn enough to raise PE). However, purchase intention consolidates only when autonomy and competence gains are clear, mirroring the PE→(PA/PC)→PI mechanism and the non‑supported IC serial chains in the quantitative model.

#### 5.2.6. Theme 6: Context & safety constraints: Infrastructure, governance, and norms.

This cross‑cutting theme appeared in all 26 interviews. Participants repeatedly emphasized that infrastructure quality, regulatory clarity/enforcement, and the mobility ecosystem (parking supply, service/repair access, theft/insurance) condition whether performance gains translate into felt autonomy and competence. Where protected lanes and clear parking rules existed, riders described “gliding” routines and predictable door‑to‑door times. Where roads were uneven, traffic norms aggressive, or rules ambiguous, the same riders reported fragile autonomy, confidence, and time advantages collapsed unpredictably.

“*On the coastal lanes it feels like gliding; in the center, cobblestones and parked cars break the flow. Some days you’re fast, other days the time advantage disappears*.” (P08)

“*After the municipality put scooter racks and signage near the tram stops, conflicts dropped. I’m more comfortable locking it and walking away; before that I was always expecting an argument.*” (P12)

“*I don’t leave it outside unless there’s a camera; theft risk is real, and insurance is pricey. For work days or heavy rain, I switch to rental or bus ownership only makes sense when the ecosystem protects the time gain*.” (P06)

Context acts as a boundary condition: supportive infrastructure and governance amplify the PE → PA/PC → PI chain by making time savings predictable and confidence durable; adverse conditions dampen it by injecting risk and variability. Decisions about ownership vs rental are often context‑responsive hedges against uncertainty in total cost (repairs, theft, weather), consistent with participants’ pragmatic emphasis on value preservation rather than novelty.

## 6. Discussion

This study explains private e-scooter purchase intention by linking value cues to PE and then to the motivational resources that make ownership feel feasible in everyday travel. PLS-SEM identifies the supported pathways; interviews explain why those pathways appear and where they weaken. The evidence indicates a process in which upstream cues shape usefulness beliefs, which are then associated with purchase intention mainly through perceived control and capability in urban settings. Table 10 connects the path estimates with interview themes, so the coefficients can be interpreted as a mechanism rather than as isolated effects..

**Table 10:** Joint display: Integration of PLS‑SEM paths with qualitative themes.

Path	Quan. Evidence(effect size)	Qual. theme (explanatory lens)	Representative quotation ID*	Integration judgment	Explanatory insight / boundary condition
**HM → PE**	β + , sig. (medium)	Enjoyment raises usefulness when time predictability is credible	[P05], [P12]	Confirm	Hedonic cues translate into PE only if ride quality/range reduce uncertainty (“time as currency”).
**EF → PE**	β + , sig. (medium)	Future value is conditional on infrastructure, safety, and availability	[P03], [P18]	Confirm (contingent)	Favorable LCAs and diffusion cues lift expectations, but gaps in lanes/parking/regulation discount EF.
**LPS → PE**	β + , sig. (small)	Price salience returns when TCO is opaque or downtime is likely	[P07], [P21]	Explain (attenuated)	Lower price focus helps, yet repair access/battery health/insurance risk re‑activate sacrifice, capping effect size.
**PE → PA**	β + , sig. (medium–large)	Schedule control and door‑to‑door proximity drive felt autonomy	[P02], [P14]	Confirm	Predictable access and near‑destination parking convert utility into control.
**PE → PC**	β + , sig. (medium–large)	Routine mastery (micro‑skills, route knowledge) builds competence	[P09], [P16]	Confirm	Short learning curve + safety cues lower performance risk and increase self‑efficacy.
**PE → PI (direct)**	β + , sig. (small)	Utility must be consistent across days, not episodic	[P01]	Explain (modest)	Users need reliability, not peak specs; inconsistency weakens the direct channel.
**PA → PI**	β + , sig. (small–medium)	“No waiting” and route freedom tip the decision	[P10]	Confirm	Autonomy matters more where transit wait times and parking frictions are salient.
**PC → PI**	β + , sig. (medium)	Confidence reduces perceived risk and unlocks commitment	[P08], [P20]	Confirm (stronger)	Hostile traffic or vague rules erode PC; supportive design/policy stabilizes intention.
**PE → IC**	β + , sig. (medium)	Novelty sparks trial and feature exploration	[P06]	Partial	Exploration rises with PE, but it remains a trial‑phase response.
**IC → PI**	β + , sig.(small)	Identity ambivalence; novelty insufficient for purchase	[P11], [P19]	Confirm(weak)	IC has a small directassociation with PI, butdoes not carry the serialmediation chains.Purchase remainspragmatic and risk aware.
**HM → PE → PA → PI (serial)**	sig. indirect	Hedonics matter through utility → control	[P05], [P02]	Confirm	Affective value converts to purchase via PE → PA.
**HM/EF/LPS → PE → PC → PI (serial)**	sig. indirect	Utility builds capability, then intention	[P09], [P20]	Confirm	Competence is the workhorse channel.
**HM/EF/LPS → PE → IC → PI (serial)**	n.s.	Trial ≠ purchase under utility‑first logic	[P06], [P11]	Explain (not supported)	IC energizes exploration but does not carry ownership decisions in this context.

The findings support a utility-first decision logic. HM, EF, and LPS strengthen PE, while PE is translated into PI chiefly through PA and PC rather than IC. This pattern aligns with acceptance research that positions PE as the proximal cognitive driver of intention [8,22] and with evidence that enjoyment elevates usefulness appraisals by increasing engagement and perceived task fit [23,24]. The interviews add an important qualification: hedonic cues matter when the practical promise is credible. Participants repeatedly framed time as a scarce resource, which explains why HM works through PE and why PE has pronounced paths to PA and PC [9,10]. Table 10 shows where the qualitative evidence corroborates the coefficients, introduces boundary conditions, or explains why PA and PC carry the conversion from PE to PI..

The EF → PE path indicates that future-oriented beliefs can raise perceived performance. Life-cycle evidence on durability and logistics supports the view that sustainability expectations may strengthen utility beliefs [27], while city-level evidence suggests that e-scooter access can create perceived convenience value [13]. This interpretation requires caution because EF is a broad forward-looking construct that may combine sustainability expectations, technological maturation, and market diffusion beliefs. The coefficient should therefore be read as evidence of a general future-oriented evaluation rather than as the effect of one specific expectation dimension. The interviews show that expectations about range, safety, availability, and regulation are discounted when infrastructure is fragmented or enforcement is unclear. This credibility filter helps explain the moderate EF → PE effect despite favorable narratives about sustainability and diffusion [26,28].

The LPS → PE relationship is positive but modest, which is consistent with a value-evaluation account in an emerging-market context. When price salience decreases, evaluative attention shifts from monetary sacrifice toward functional benefits, which should raise PE [29,30]. The qualitative evidence, however, indicates that this shift is fragile. TCO ambiguity, including battery health uncertainty, parts availability, and repair downtime, together with perceived risk premiums in everyday use, can reactivate price focus, particularly among first-time buyers. Supply-side analyses reinforce this interpretation by showing that operations and leasing dominate cost structures and that swapping logistics impose binding constraints that ultimately surface as reliability and downtime concerns for users [17,18]. In practical terms, lower price sensitivity facilitates higher PE only when performance signals are credible and when the ownership cost envelope is sufficiently transparent for consumers to evaluate value with confidence.

The strong PE → PA and PE → PC relationships, together with the modest direct PE → PI effect, are consistent with SDT: performance expectations appear to foster felt control and competence, and these need-relevant states carry intention [73]. The interviews provide a concrete behavioral interpretation of this mechanism. Participants described schedule control, including reduced waiting and point-to-point travel, and routine mastery, including micro-skill development and situational awareness in traffic, as the levers that make purchase feel viable, especially when parking proximity and protected links reduce day-to-day friction. At the same time, the qualitative evidence indicates clear boundary conditions. Where infrastructure is perceived as hostile or enforcement is perceived as inconsistent, competence becomes fragile, and the direct translation from PE into PI weakens, consistent with the quantitative pattern and echoing accessibility and equity findings documented in shared systems [15,34].

The unsupported serial mediation paths through IC clarify the role of innovativeness in this purchase context. Prior evidence indicates that hedonic and innovative routes can be influential in sharing markets, where trial is low-commitment and novelty is easier to express behaviorally [11,12,27]. In the present model, PE significantly increased IC with a medium effect size, and IC had a small direct association with PI. Yet IC did not transmit the effects of HM, EF, or LPS to PI. The interviews explain this pattern: novelty can spark trial and feature exploration, but ownership decisions depend more on reliable gains in control and capability. Participants also described image ambivalence, weather exposure, theft concerns, and uneven lanes, which reduced the purchase relevance of innovativeness. Consistent with Table 10, IC appears to support exploration, whereas PA and PC carry the main conversion from performance appraisal to ownership intention..

The quantitative and qualitative strands point to the same pattern. Consumers first evaluate whether e-scooter ownership offers credible everyday utility. PI is then associated mainly with autonomy and competence, while this interpretation depends on supportive conditions such as infrastructure quality, rule clarity, and after-sales access. This convergence suggests that the model captures a practical mechanism for understanding ownership interest in this setting.

## 7. Contributions and implications

This study advances micromobility adoption research by demonstrating that a mediated mechanism better explains private purchase intention than do direct motive-to-intention links. Across PLS-SEM and pattern-matching analyses of 26 interviews, the evidence converges on a central pivot: PE as a cognitive value appraisal that translates upstream cues into downstream commitment. HM, EF, and LPS primarily matter insofar as they strengthen PE, while interview accounts clarify why credibility of practical benefit and perceived control dominate the purchase narrative.

The findings refine the adoption logic by specifying how PE becomes consequential. Rather than operating mainly through novelty-oriented exploration, PE is transmitted to PI chiefly through PA and PC, which positions autonomy and competence as the critical hinge between appraisal and commitment. This distinction also clarifies stage specificity: novelty may support trial and early exploration, yet enduring purchase intention rests on predictable utility and rapid mastery that sustain PA and PC.

Methodologically, the study strengthens inference by triangulating serial mediation testing in PLS-SEM with qualitative pattern matching. The quantitative results establish the structure and relative strength of the pathways, while the interviews provide contextual explanations and boundary conditions that indicate when the mechanism is likely to hold, and when it is discounted.

The findings offer actionable implications for firms and city managers, particularly in relation to younger urban consumers in emerging-market contexts. For firms, the evidence suggests prioritizing value credibility and mastery over novelty positioning when addressing this segment.. Product and service design for this segment should emphasize reliability, safety, and service continuity, supplemented by risk-reducing assurances such as warranty visibility, fast repair access, and practical onboarding that accelerates competence. Pricing actions are likely to be most effective when younger urban consumers can evaluate value with confidence, which underscores the managerial importance of transparent total cost of ownership and credible performance signals.

For policy and governance, the findings should be interpreted within the study context. Among younger urban consumers in an emerging-market setting, infrastructure quality, rule clarity, parking supply, theft risk, and service access shape whether PE is viewed as credible and whether PA and PC can be sustained in everyday travel.. Public-private coordination that improves lanes, parking, and repair coverage amplifies the returns on firm-level investments. Where conditions remain weak or uncertain, rental and subscription options can function as transitional arrangements that lower commitment barriers while the ecosystem matures.

The overall contributions are summarized in Table 11, which links theoretical refinement, empirical support, boundary conditions, and managerial actions in a compact map.

**Table 11:** Contribution map: Theoretical, managerial, and contextual insights.

Insight	Theoretical contribution	Evidence (PLS-SEM + interviews)	Boundary conditions and managerial move
PE is the pivotal appraisal	Frames adoption as a mechanism driven by cognitive value appraisal, not a predictor list	HM, EF, LPS primarily raise PE; indirect effects dominate; interviews stress predictable utility and time savings	Credibility depends on infrastructure and rule clarity; demonstrate reliability and access continuity with concrete proof points
PA and PC convert PE into PI	Specifies an SDT-consistent conversion pathway from appraisal to commitment	Strong PE → PA/PC; substantive PA/PC → PI; direct PE → PI comparatively small; interviews emphasize control and mastery	Enable autonomy and competence via onboarding and service support; improve lanes and parking to reduce friction
EF and LPS are conditional inputs to PE	Explains forward-looking beliefs and value evaluation as contingent, not universal, drivers	EF → PE moderate; LPS → PE small; interviews show discounting under unclear rules and TCO ambiguity	Reduce EF discounting with governance clarity and ecosystem readiness; make TCO calculable via warranty, repair speed, and transparent service pricing
IC supports exploration, but is not themain purchasepathway	Distinguishes trial and exploration from ownership commitment	PE → IC is medium;IC → PI is small;IC-based serial chainsare not supported;interviews framepurchase as pragmaticand risk aware	Use short trials for high-IC segments; for the mainstream, emphasize predictability, control, and competence building

## 8. Limitations and future research directions

This study has several boundaries of inference. First, the serial mediation results are theory-consistent but are estimated from cross-sectional survey data, so temporal ordering is assumed rather than observed. The proposed mechanism should therefore be interpreted as a process-consistent account rather than as a verified causal sequence. Longitudinal, panel, diary, or event-based designs would be better suited to test whether PE develops before autonomy and competence and whether these states precede purchase intention over time.

Second, the outcome is PI rather than realized purchase, leaving the intention-behavior gap untested; future work that links surveys to registrations, transaction data, or follow-up panels would strengthen behavioral validity. Third, constructs are self-reported; although bias checks were satisfactory, incorporating telemetry, service records, or objective performance indicators would further reduce common-method concerns. Finally, EF was operationalized as a broad future-oriented reflective construct covering sustainability expectations, technological maturation, and market diffusion, which may mask differences across these facets. The EF coefficient should therefore be interpreted as the effect of a general future-oriented evaluation rather than as evidence for any single expectation dimension. LPS was also modeled reflectively, but it may still capture both trait-like and situational responses to monetary sacrifice in this context. Future research may separate these dimensions more explicitly and use experimental manipulations of price salience and assurance cues to isolate their effects more precisely.

The framework is deliberately parsimonious. It centers on PE and SDT-related needs and therefore omits constructs that may be influential in dense urban settings, such as perceived risk, social influence, and habit [74]. Adjacent digital-platform research also points to intermediate states such as perceived benefits, satisfaction, attachment, identification, and belongingness as potentially relevant mechanisms in post-adoption behavior [75–77]. Future research may test whether these constructs add explanatory power in private micromobility ownership contexts. The limited role of IC in the present model may also conceal heterogeneity; mixture models or latent-class SEM could test whether specific segments exhibit novelty-driven purchasing.

The data come from a young, student-dominated urban sample in Turkey, which limits the breadth of generalization. The reported mechanism should therefore be interpreted primarily in relation to this segment rather than to all private e-scooter consumers. Age, employment status, commuting routines, household responsibilities, and established car ownership may alter the relative importance of performance expectancy, autonomy, competence, price sensitivity, and safety-related concerns. The findings may therefore not transfer directly to older adults, full-time workers, parents, or more car-dependent populations. The Turkish setting may also limit transferability. As an emerging-market context, Turkey combines uneven micromobility infrastructure, evolving regulatory and enforcement conditions, and high consumer attention to cost and value. These features may strengthen the practical salience of performance expectancy, ownership frictions, and price-related evaluations relative to settings with more mature infrastructure and governance**.**

Although PLS-SEM was selected for prediction and mediation, robustness checks using CB-SEM or Bayesian SEM, as well as tests of non-linearity, would be valuable. Future research should also incorporate contextual enablers and constraints, including objective infrastructure measures, enforcement intensity, theft risk, and supply-side assurance levers such as retail channel quality and warranty access, to identify when the utility-to-autonomy and competence pathway is strengthened or disrupted.

## 9. Conclusion

This study contributes to micromobility research in three ways. First, it shifts the focus from direct motive-to-intention explanations to a mechanism-based account of private e-scooter purchase intention. Prior work has largely emphasized shared-use adoption, continuance, or direct effects of motives and evaluations on intention [9–12,45,46]. In contrast, our findings show that hedonic motivation, future expectations, and low price sensitivity matter mainly because they strengthen performance expectancy, which then increases purchase intention. Second, the study shows that performance expectancy is translated into intention primarily through autonomy and competence, which extends technology-acceptance research by identifying the motivational route through which performance beliefs become commitment [22,32]. Third, the findings indicate that innovative consumption plays a more limited role than the literature on novelty-oriented adoption might suggest [11,12]. This points to a utility-first ownership logic in which consumers prioritize reliable everyday value over experimentation.

The results support a utility-first logic. HM, EF, and LPS strengthen PE, but purchase intention is carried mainly through autonomy and competence rather than novelty-oriented exploration. The qualitative findings show when this mechanism holds and when it weakens. Infrastructure quality, regulatory clarity, and after-sales access shape whether PE becomes durable autonomy and competence in everyday travel. For firms targeting younger urban consumers in emerging-market contexts, the main implication is to make performance credibility visible through reliability, service continuity, warranty support, and transparent ownership cost. For policymakers in similar contexts, safer infrastructure and clearer governance can strengthen confidence in daily use..

## Supporting information

S1 Fig Structural model with standardized path coefficients (PLS-SEM). (TIF)

S1 File Supplementary materials. This file includes the measurement scales, common method bias assessment, measurement model diagnostics, qualitative participant characteristics, semi-structured interview guide, structural model multicollinearity assessment, and detailed qualitative codebook.
(DOCX)

S1 Data Anonymized quantitative dataset. (CSV)

## References

[1] North American Bikeshare & Scootershare Association (NABSA). 2024 Shared Micromobility State of the Industry Report [Internet]. 2025 [cited 2025 Apr 28]. Available from: https://nabsa.net/2025/08/07/2024industryreport/

[2] International Transport Forum (ITF). Greener Micromobility [Internet]. 2024 [cited 2025 Apr 29]. Available from: https://www.itf-oecd.org/greener-micromobility

[3] Micro-Mobility for Europe (MMfE). Incident Data 2024 [Internet]. 2025 [cited 2025 Apr 29]. Available from: https://micromobilityforeurope.eu/incident-data-2024-2/

[4] Ville de Paris. Fin des trottinettes en libre service à Paris le 31 août 2023 [Internet]. 2023 Apr 3 [cited 2025 May 22]. Available from: https://www.paris.fr/pages/pour-ou-contre-les-trottinettes-en-libre-service-23231

[5] Reuters. Italy to enforce helmets, insurance for e-scooter riders after accidents [Internet]. 2024 [cited 2025 Aug 29]. Available from: https://www.reuters.com/world/europe/italy-enforce-helmets-insurance-e-scooter-riders-after-accidents-2024-11-20/

[6] Bird. Bird successfully emerges from bankruptcy as a stronger company and will operate as the global anchor brand of newly established Third Lane Mobility Inc [Internet]. 2024 [cited 2025 May 11]. Available from: https://www.bird.co/blog/bird-successfully-emerges-from-bankruptcy-as-a-stronger-company-and-will-operate-as-the-global-anchor-brand-of-newly-established-third-lane-mobility-inc/

[7] Indah DR, Putra AP, Firdaus MA. Analysis of User Acceptance Using UTAUT2 Model in KAI Access Application. J Teknol Inf Dan Pendidik. 2023;15(2):85–95. doi: 10.24036/jtip.v15i2.553

[8] Venkatesh V, Thong JYL, Xu X. Consumer Acceptance and Use of Information Technology: Extending the Unified Theory of Acceptance and Use of Technology1. MIS Q. 2012;36(1):157–78. doi: 10.2307/41410412

[9] Fil MM, Dirsehan T. Eco? No, ego-scooters: steering towards time-saving trails in urban mobility. Res Transp Bus Manag. 2024;56:101190. doi: 10.1016/j.rtbm.2024.101190

[10] Arı E, Yılmaz V. Investigating the factors affecting electric scooter usage behavior with a proposed structural model. Res Transp Bus Manag. 2024;56:101164. doi: 10.1016/j.rtbm.2024.101164

[11] Flores PJ. What motivates consumers to adopt controversial green mobility innovations? The case of shared e-bikes and e-scooters. Technol Forecast Soc Change. 2024;208:123694. doi: 10.1016/j.techfore.2024.123694

[12] Flores PJ, Jansson J. Being innovative, fun, and green? Hedonic and environmental motivations in the use of green innovations. J Mark Manag. 2022;38(17–18):1907–36. doi: 10.1080/0267257X.2022.2062426

[13] Kim K, McCarthy DM. Wheels to meals: measuring the impact of micromobility on restaurant demand. J Mark Res. 2024;61(1):128–42. doi: 10.1177/00222437231179021

[14] Skiera B, Yan SY, Daxenberger J, Dombois M, Gurevych I. Using information-seeking argument mining to improve service. J Serv Res. 2022;25(4):537–48. doi: 10.1177/10946705221110845

[15] Karimi S, Kluger R. Urban micromobility and social equity: an investigation through the lens of shared e-scooter rebalancing. Res Transp Bus Manag. 2025;61:101418. doi: 10.1016/j.rtbm.2025.101418

[16] Campisi T, Kuskapan E, Çodur MY, Dissanayake D. Exploring the influence of socio-economic aspects on the use of electric scooters using machine learning applications: a case study in the city of Palermo. Res Transp Bus Manag. 2024;56:101172. doi: 10.1016/j.rtbm.2024.101172

[17] Leurent F. What is the value of swappable batteries for a shared e-scooter service? Res Transp Bus Manag. 2022;45(Pt C):100843. doi: 10.1016/j.rtbm.2022.100843

[18] Abt R, Krauss K. Cost and viability estimation of shared mobility services. Res Transp Bus Manag. 2025;62:101436. doi: 10.1016/j.rtbm.2025.101436

[19] Raji M, Magnusson P, Martirosyan Y. How emerging-market brands can overcome a weak country image. Int Mark Rev. 2025;42(1):128–48. doi: 10.1108/IMR-02-2024-0051

[20] Karadayı-Usta S. Achieving sustainability via micromobility solutions in the hospitality industry: a risk analysis case study with internal stakeholders’ perspectives. Res Transp Bus Manag. 2025;60:101374. doi: 10.1016/j.rtbm.2025.101374

[21] Mehrabian A, Russell JA. The basic emotional impact of environments. Percept Mot Skills. 1974;38(1):283–301. doi: 10.2466/pms.1974.38.1.283 4815507

[22] Davis FD. Perceived Usefulness, Perceived Ease of Use, and User Acceptance of Information Technology. MIS Q. 1989;13(3):319–40. doi: 10.2307/249008

[23] Agarwal R, Karahanna E. Time Flies When You’re Having Fun: Cognitive Absorption and Beliefs About Information Technology Usage1. MIS Q. 2000;24(4):665–94. doi: 10.2307/3250951

[24] van der Heijden H. User Acceptance of Hedonic Information Systems1. MIS Q. 2004;28(4):695–704. doi: 10.2307/25148660

[25] Vroom VH. Work and motivation. New York: John Wiley & Sons; 1964.

[26] Eccles JS, Wigfield A. Motivational beliefs, values, and goals. Annu Rev Psychol. 2002;53:109–32. doi: 10.1146/annurev.psych.53.100901.135153 11752481

[27] Baumgartner C, Helmers E. Life cycle assessment of electric kick scooters: consolidating environmental impact quantification and concluding climate-friendly use options. Environ Sci Eur. 2024;36:96. doi: 10.1186/s12302-024-00920-x

[28] Rogers EM. Diffusion of innovations. 5th ed. New York: Free Press; 2003.

[29] Zeithaml VA. Consumer perceptions of price, quality, and value: a means–end model and synthesis of evidence. J Mark. 1988;52(3):2–22. doi: 10.1177/002224298805200302

[30] Dodds WB, Monroe KB, Grewal D. Effects of price, brand, and store information on buyers’ product evaluations. J Mark Res. 1991;28(3):307–19. doi: 10.2307/3172866

[31] Chien YS, Liang JK, Lu CC. Investigating factors that influence the intention to use electric scooter sharing in a market with multiple service providers: a combined UTAUT2 and brand attitude perspective. Transp Res Rec. 2024;2678(4):25–39. doi: 10.1177/03611981231185142

[32] Ryan RM, Deci EL. Self-determination theory: basic psychological needs in motivation, development, and wellness. New York: Guilford Press; 2017.

[33] Bandura A. Self-efficacy: the exercise of control. New York: W. H. Freeman; 1997.

[34] Chou K-Y, Paulsen M, Møller M, Fjendbo Jensen A. Cyclists’ mobility and subjective safety in shared urban spaces - a simulator study. Transp Res Part F Traffic Psychol Behav. 2025;115:103321. doi: 10.1016/j.trf.2025.07.031

[35] Midgley DF, Dowling GR. Innovativeness: the concept and its measurement. J Consum Res. 1978;4(4):229–42. doi: 10.1086/208701

[36] Agarwal R, Prasad J. A conceptual and operational definition of personal innovativeness in the domain of information technology. Inf Syst Res. 1998;9(2):204–15. doi: 10.1287/isre.9.2.204

[37] Roehrich G. Consumer innovativeness: concepts and measurements. J Bus Res. 2004;57(6):671–7. doi: 10.1016/S0148-2963(02)00311-9

[38] Moon JW, Kim YG. Extending the TAM for a World Wide Web context. Inf Manage. 2001;38:217–30. doi: 10.1016/S0378-7206(00)00061-6

[39] Eccarius T, Lu C-C. Adoption intentions for micro-mobility – Insights from electric scooter sharing in Taiwan. Transp Res Part D Transp Environ. 2020;84:102327. doi: 10.1016/j.trd.2020.102327

[40] Kopplin CS, Brand BM, Reichenberger Y. Consumer acceptance of shared e-scooters for urban and short-distance mobility. Transportation Research Part D: Transport and Environment. 2021;91:102680. doi: 10.1016/j.trd.2020.102680

[41] Kim H-W, Chan HC, Gupta S. Value-based Adoption of Mobile Internet: An empirical investigation. Decis Support Syst. 2007;43(1):111–26. doi: 10.1016/j.dss.2005.05.009

[42] An Z, Mullen C, Guan X, Ettema D, Heinen E. Shared micromobility, perceived accessibility, and social capital. Transportation (Amst). 2026;53(2):1025–60. doi: 10.1007/s11116-024-10521-5 41810330 PMC12968100

[43] Nagdev K, Rajesh A. Extending intention to use toward postadoption behavior—Conceptualizing actual usage for information technology-enabled banking services. Technol Mind Behav. 2024;5(2):98–114. doi: 10.1037/tmb0000132

[44] Hwang GJ, Wu PH, Chen CC, Tu NT. Effects of an augmented reality based educational game on students’ learning achievements and attitudes in real world observations. Interact Learn Environ. 2015;24(8):1895–906. doi: 10.1080/10494820.2015.1057747

[45] Çallı L. Value centric analysis of user adoption for sustainable urban micro mobility transportation through shared e scooter services. Sustain Dev. 2024;32(6):6408–33. doi: 10.1002/sd.3032

[46] Marroquín-Ciendúa F, Medina-Labrador M, Hurtado Méndez LC, Mora Hernández LP, Puentes Guzmán CN. Use of UTAUT for analyzing the acceptance and use of electric scooters in the public transport system. Urban Plan Transp Res. 2025;13(1):65–82. doi: 10.1080/21650020.2025.2458548

[47] David L, Weinstein N. Using technology to make learning fun: technology use is best made fun and challenging to optimize intrinsic motivation and engagement. Eur J Psychol Educ. 2024;39:1441–63. doi: 10.1007/s10212-023-00734-0

[48] Thai NH, Quan TM. Factors Influencing Individual Consumers’ Intentions To Purchase Electric Vehicles In Urban Hanoi. TP. 2024;19(4):17–29. doi: 10.20858/tp.2024.19.4.02

[49] Zhao H, Furuoka F, Rasiah R. The Influence of Psychological Factors on Consumer Purchase Intention for Electric Vehicles: Case Study from China: Integrating the Necessary Condition Analysis Methodology from the Perspective of Self-Determination Theory. WEVJ. 2024;15(8):331. doi: 10.3390/wevj15080331

[50] Adnan N. Exploring the future: A meta-analysis of autonomous vehicle adoption and its impact on urban life and the healthcare sector. Transp Res Interdiscip Perspect. 2024;26:101110. doi: 10.1016/j.trip.2024.101110

[51] Parmar J, Delle Site P, Dissanayake D. Understanding individuals’ intentions to use shared electric mobility using a structural equation modelling approach. SSRN [Preprint]. 2025 [cited 2025 Dec 27]. Available from: doi: 10.2139/ssrn.5139582

[52] Huang F-H. Factors Influencing Sustained Use of Shared E-Scooter Services in Urban Taiwan. Promet Traffic Transp. 2024;36(5):902–21. doi: 10.7307/ptt.v36i5.616

[53] Dias G, Ribeiro P, Arsenio E. Determinants of shared e scooter usage and their policy implications: findings from a survey in Braga, Portugal. Eur Transp Res Rev. 2024;16:20. doi: 10.1186/s12544-024-00642-4

[54] Sugihara C, Hardman S. Electrifying California fleets: Investigating light-duty vehicle purchase decisions. Transp Res Interdiscip Perspect. 2022;13:100532. doi: 10.1016/j.trip.2021.100532

[55] Walton T, Torija AJ, Hughes RJ, Elliott AS. Evaluation of auditory alerting systems for safe electric scooter operations. Sci Rep. 2025;15(1):3424. doi: 10.1038/s41598-024-80975-1 39870658 PMC11772828

[56] Kurz J, Efendić E, Goukens C. Pricey therefore good? Price affects expectations, but not quality perceptions and liking. Psychol Mark. 2023;40:1115–29. doi: 10.1002/mar.21799

[57] Blake T, Nosko C, Tadelis S. Consumer Heterogeneity and Paid Search Effectiveness: A Large-Scale Field Experiment. Econometrica. 2015;83(1):155–74. doi: 10.3982/ecta12423

[58] Vidal-Silva C, Sánchez-Ortiz A, Serrano-Malebrán J, Arriagada V, Flores M, Godoy M, et al. Social influence, performance expectancy, and price value as determinants of telemedicine services acceptance in Chile. Heliyon. 2024;10(5):e27067. doi: 10.1016/j.heliyon.2024.e27067 38562504 PMC10982984

[59] Bieliński T, Czuba T, Dopierała Ł, Tarkowski M. Electric bike sharing: price sensitivity and pricing preferences. Res Transp Bus Manag. 2024;56:101163. doi: 10.1016/j.rtbm.2024.101163

[60] Askari S, Javadinasr M, Peiravian F, Khan NA, Auld J, Mohammadian AK. Loyalty toward shared e-scooter: Exploring the role of service quality, satisfaction, and environmental consciousness. Travel Behav Soc. 2024;37:100856. doi: 10.1016/j.tbs.2024.100856

[61] Cubells J, Miralles-Guasch C, Marquet O. E scooter and bike share route choice and detours: modelling the influence of built environment and sociodemographic factors. J Transp Geogr. 2023;111:103664. doi: 10.1016/j.jtrangeo.2023.103664

[62] Lin C, Xue X, Zhu Z, Luo Y, Song R. Factors related to the intention of choosing shared E-scooters for metro transfer: A survey study integrating weather perception into satisfaction evaluation from Changsha. PLoS One. 2024;19(9):e0309953. doi: 10.1371/journal.pone.0309953 39250487 PMC11383246

[63] Shmueli G, Sarstedt M, Hair JF, Cheah JH, Ting H, Vaithilingam S. Predictive model assessment in PLS-SEM using PLSpredict. Eur J Mark. 2019;53(11):2322–47. doi: 10.1108/EJM-02-2019-0189

[64] Hair JF, Hult GTM, Ringle CM, Sarstedt M. A primer on partial least squares structural equation modeling (PLS-SEM). 3rd ed. Sage; 2022.

[65] Nunnally JC, Bernstein IH. Psychometric theory. 3rd ed. McGraw-Hill; 1994.

[66] Fornell C, Larcker DF. Evaluating structural equation models with unobservable variables and measurement error. J Mark Res. 1981;18(1):39–50. doi: 10.1177/002224378101800104

[67] Henseler J, Ringle CM, Sarstedt M. Testing measurement invariance of composites using partial least squares. Int Mark Rev. 2016;33(3):405–31. doi: 10.1108/IMR-09-2014-0304

[68] Hair JF, Risher JJ, Sarstedt M, Ringle CM. When to use and how to report the results of PLS-SEM. EBR. 2019;31(1):2–24. doi: 10.1108/ebr-11-2018-0203

[69] Braun V, Clarke V. Using thematic analysis in psychology. Qual Res Psychol. 2006;3(2):77–101. doi: 10.1191/1478088706qp063oa

[70] Braun V, Clarke V. Reflecting on reflexive thematic analysis. Qual Res Sport Exerc Health. 2019;11(4):589–97. doi: 10.1080/2159676x.2019.1628806

[71] McHugh ML. Interrater reliability: the kappa statistic. Biochem Med (Zagreb). 2012;22(3):276–82. doi: 10.11613/bm.2012.031 23092060 PMC3900052

[72] Cohen J. Statistical power analysis for the behavioral sciences. 2nd ed. New York: Routledge; 1988.

[73] Deci EL, Ryan RM. The “what” and “why” of goal pursuits: human needs and the self-determination of behavior. Psychol Inq. 2000;11(4):227–68. doi: 10.1207/S15327965PLI1104_01

[74] Zhao AP, Li S, Alhazmi M, Bao Z, Cheng X. Psychological insights for electric vehicles. Int J Electr Power Energy Syst. 2025;171:110931. doi: 10.1016/j.ijepes.2025.110931

[75] Pang H, Ruan Y. Disentangling composite influences of social connectivity and system interactivity on continuance intention in mobile short video applications: The pivotal moderation of user-perceived benefits. J Retail Consum Serv. 2024;80:103923. doi: 10.1016/j.jretconser.2024.103923

[76] Pang H, Zhang K. Determining influence of service quality on user identification, belongingness, and satisfaction on mobile social media: Insight from emotional attachment perspective. J Retail Consum Serv. 2024;77:103688. doi: 10.1016/j.jretconser.2023.103688

[77] Pang H, Zhang K. How multidimensional benefits determine cumulative satisfaction and eWOM engagement on mobile social media: Reconciling motivation and expectation disconfirmation perspectives. Telematics Inform. 2024;93:102174. doi: 10.1016/j.tele.2024.102174

