# Supplementary figures and images for: Utility over novelty: How performance expectancy converts hedonic motivation, future expectations, and price sensitivity into private e-scooter purchase intention
Source: PLoS One. 2026 Jul 6;21(7):e0341194. doi: 10.1371/journal.pone.0341194 (PMC13336187; doi:10.1371/journal.pone.0341194)

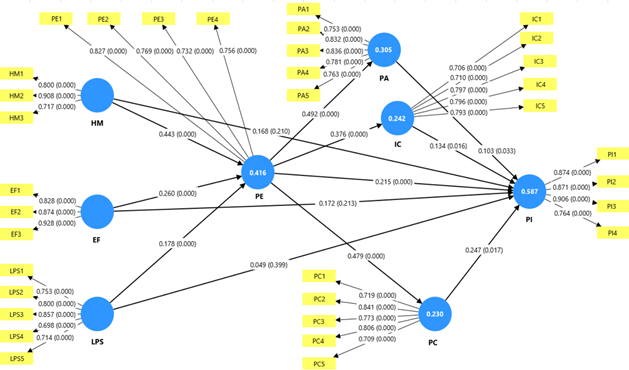

Supplement: S1 Fig — (TIF) [file pone.0341194.s001.tif]
