# Supplementary material for: Utility over novelty: How performance expectancy converts hedonic motivation, future expectations, and price sensitivity into private e-scooter purchase intention
Source: PLoS One. 2026 Jul 6;21(7):e0341194. doi: 10.1371/journal.pone.0341194 (PMC13336187; doi:10.1371/journal.pone.0341194)
Supplement: S1 File — This file includes the measurement scales, common method bias assessment, measurement model diagnostics, qualitative participant characteristics, semi-structured interview guide, structural model multicollinearity assessment, and detailed qualitative codebook. (DOCX) [file pone.0341194.s002.docx]

**Utility Over Novelty: How Performance Expectancy Converts Hedonic Motivation, Future Expectations, and Price Sensitivity into Private E-Scooter Purchase Intention**

**Supporting information**

**Table** **S1.** Measurement Scales, Items, and References

| Consctruct | Items | Reference |
| --- | --- | --- |
| Purchase Intention (PI) | |  |
| PI1 | I am interested in e-scooters and have been paying attention to them recently. | *Dodds et al., (1991), Xie et al., (2022).* |
| PI2 | If there is a suitable model, I will consider buying it. |  |
| PI3 | I would like to recommend people I know to buy an e-scooter. |  |
| PI4 | I support the purchase of e-scooters. |  |
| ****Performance Expectancy (PE)**** | |  |
| PE1 | Using an e-scooter would enhance my travel effectiveness. | *Lee et al., (2019).* |
| PE2 | Using an e-scooter would increase my productivity in daily travel. |  |
| PE3 | Using an e-scooter would enhance my travel performance. |  |
| PE4 | I would find an e-scooter useful for my mobility needs. |  |
|  | Using an e-scooter would help me accomplish my trips more efficiently. |  |
| ****Hedonic Motivation (HM)**** | |  |
| HM1 | Using e-scooter would be fun. | *Kapser & Abdelrahman, (2020).* |
| HM2 | Using e-scooter would be enjoyable. |  |
| HM3 | Using e-scooter would be very entertaining |  |
| ****Lack of Price Sensitivity (LPS)**** | |  |
| **LPS1** | I would not mind paying more to use an e-scooter. | *Foroughi et al., (2023).* |
| **LPS2** | I would not mind spending a lot of money to use an e-scooter. |  |
| **LPS3** | I would be less willing to pay for e-scooters if I thought them to be high in price. *(reverse coded)* |  |
| **LPS4** | If using e-scooters is likely to be more expensive than conventional transport, that would not matter to me. |  |
| **LPS5** | A really great e-scooter option would be worth paying a lot of money for. |  |
| Expectation of ES in the future (EF) | |  |
| EF1 | The shift from conventional transport modes to e-scooters is a growing global trend. | *[Thiel et al. (2012)](https://www.sciencedirect.com/science/article/pii/S1361920919308296?via%3Dihub#b0415); Kwon et al., (2020).* |
| EF2 | Future safety standards for e-scooters will be fully considered and effectively implemented. |  |
| EF3 | Future battery technology for e-scooters will be stable, and replacement costs will be lower. |  |
| Perceived Competence (PC) | |  |
| PC1 | When considering personal transport, I will prioritize e-scooters with advanced technology and reliable performance. | *Franke & Krems, (2013).* |
| PC2 | I intend to purchase an e-scooter because I believe I can fully master its riding technology. |  |
| PC3 | I feel confident and not troubled about the maintenance and use of e-scooters. |  |
| PC4 | I am confident in purchasing an e-scooter because I believe I can handle possible technical challenges. |  |
| PC5 | When considering purchasing an e-scooter, I will thoroughly research its technological performance. |  |
| Perceived Autonomy (PA) | |  |
| PA1 | When making a personal transport purchase decision, I am more inclined to base it on my own needs rather than external pressure. | *Charng et al., (1988); Zhao et al., (2024).* |
| PA2 | I feel that purchasing an e-scooter is an autonomous decision, rather than a trend-following behavior. |  |
| PA3 | When purchasing a personal transport option, I am more concerned about whether the e-scooter can meet my specific travel needs. |  |
| PA4 | When purchasing an e-scooter, I am not influenced by social opinion about them. |  |
| PA5 | I feel that purchasing an e-scooter is my own way of contributing to environmental protection. |  |
| Innovative Consumption (IC) | |  |
| IC1 | I am interested in trying novel mobility options that I have not experienced before. | *Kidwell & Jewell, (2003), Krügel & Uhl, (2022).* |
| IC2 | I like to try e-scooter models or services that are relatively new on the market and used by few people. |  |
| IC3 | I tend to purchase mobility products that integrate technology and innovation. |  |
| IC4 | I have a strong interest in e-scooters that adopt the latest technology and design. |  |
| IC5 | Purchasing an e-scooter would be a reflection of my appreciation for technological innovation. |  |

**Table S2.** Common method bias assessment: procedural remedies and statistical diagnostics.

| Assessment | What was done | Criterion | Result | Interpretation |
| --- | --- | --- | --- | --- |
| Anonymity and confidentiality | Respondents were informed that the survey was anonymous and that there were no right or wrong answers. | Procedural remedy | Applied | Reduced evaluation apprehension and social desirability bias. |
| Psychological separation of constructs | Predictor and criterion constructs were presented in separate sections of the questionnaire. | Procedural remedy | Applied | Reduced respondents’ tendency to infer relationships among constructs. |
| Item ordering | Questionnaire items were presented in mixed order where appropriate. | Procedural remedy | Applied | Reduced consistency motifs and pattern responding. |
| Full collinearity assessment | Full collinearity VIF values were examined for all latent constructs. | VIF < 3.3 | 1.284 to 2.417 | No indication of serious common method bias. |
| Marker-variable check | A theoretically unrelated marker variable was included in the assessment. | Very low or non-significant correlations | r = .062 to .118 | Additional evidence against substantial common method bias. |
| Harman’s single-factor test | Exploratory factor analysis was used to assess single-factor dominance. | First factor < 50% | 31.47% | No single factor accounted for the majority of variance. |

**Table S3.** Measurement Model: Indicator Loadings, Descriptive Statistics, and VIF Values

| Constructs | Loading | Mean | SD | VIF |
| --- | --- | --- | --- | --- |
| Expectation of ES in the future (EF) | | | | |
| EF1 | 0.828 | 2.251 | 1.179 | 2.630 |
| EF2 | 0.874 | 2.521 | 1.290 | 2.638 |
| EF3 | 0.928 | 2.393 | 1.224 | 2.637 |
| Hedonic Motivation (HM) |  |  |  |  |
| HM1 | 0.800 | 2.624 | 1.270 | 1.643 |
| HM2 | 0.908 | 2.287 | 1.119 | 2.026 |
| HM3 | 0.717 | 2.612 | 1.248 | 1.364 |
| Innovative Consumption (IC) |  |  |  |  |
| IC1 | 0.706 | 3.639 | 1.052 | 1.847 |
| IC2 | 0.710 | 3.243 | 1.212 | 1.720 |
| IC3 | 0.797 | 3.621 | 1.090 | 2.375 |
| IC4 | 0.796 | 3.408 | 1.159 | 2.185 |
| IC5 | 0.793 | 2.707 | 1.245 | 1.350 |
| Lack of Price Sensitivity (LPS) |  |  |  |  |
| LPS1 | 0.752 | 3.485 | 1.154 | 1.459 |
| LPS2 | 0.801 | 3.154 | 1.228 | 2.011 |
| LPS3 | 0.857 | 3.074 | 1.256 | 2.069 |
| LPS4 | 0.699 | 2.382 | 1.266 | 2.421 |
| LPS5 | 0.715 | 2.322 | 1.271 | 2.178 |
| Perceived Autonomy (PA) |  |  |  |  |
| PA1 | 0.752 | 3.938 | 1.026 | 1.755 |
| PA2 | 0.832 | 3.666 | 1.103 | 1.892 |
| PA3 | 0.836 | 4.027 | 1.004 | 1.744 |
| PA4 | 0.781 | 3.583 | 1.157 | 1.361 |
| PA5 | 0.764 | 3.228 | 1.230 | 1.318 |
| Perceived Competence (PC) |  |  |  |  |
| PC1 | 0.720 | 2.624 | 1.270 | 1.716 |
| PC2 | 0.842 | 2.287 | 1.119 | 2.128 |
| PC3 | 0.773 | 2.612 | 1.248 | 2.444 |
| PC4 | 0.806 | 2.577 | 1.246 | 2.577 |
| PC5 | 0.709 | 2.624 | 1.270 | 1.324 |
| Performance Expectancy (PE) |  |  |  |  |
| ﻿PE1 | 0.827 | 3.346 | 1.195 | 1.564 |
| PE2 | 0.769 | 3.976 | 0.954 | 1.690 |
| PE3 | 0.732 | 3.624 | 1.068 | 1.613 |
| PE4 | 0.756 | 3.287 | 1.119 | 1.487 |
| Purchase Intention (PI) |  |  |  |  |
| PI1 | 0.874 | 2.251 | 1.179 | 2.654 |
| PI2 | 0.871 | 2.521 | 1.290 | 2.649 |
| PI3 | 0.907 | 2.393 | 1.224 | 2.976 |
| PI4 | 0.764 | 3.263 | 1.204 | 1.616 |

**Table S4.** Sample Characteristics of Qualitative Participants (n = 26)

| ID | Age | Education | Income | Interview Duration (min) | Commute (one-way, min) | Infrastructure Access | Mode | Scooter Experience (months) |
| --- | --- | --- | --- | --- | --- | --- | --- | --- |
| P01 | 18-24 | Bachelor’s | 22.501–39.999 | 52 | 35 | Medium | Online | 7–12 |
| P02 | 25-34 | Bachelor’s | 40.000–59.999 | 58 | 40 | Low | In-person | 7–12 |
| P03 | 25-34 | Master’s | 22.501–39.999 | 49 | 30 | High | Online | 0 |
| P04 | 35-44 | Bachelor’s | ≥ 80.000 | 61 | 50 | Medium | In-person | 13–24 |
| P05 | 25-34 | Bachelor’s | 40.000–59.999 | 47 | 25 | Medium | Online | 0 |
| P06 | 18-24 | High School | 22.501–39.999 | 54 | 45 | Low | In-person | 13–24 |
| P07 | 35-44 | Master’s | 60.000–79.999 | 63 | 50 | Medium | In-person | 1–6 |
| P08 | 25-34 | Bachelor’s | 60.000–79.999 | 57 | 40 | High | In-person | 13–24 |
| P09 | 25-34 | Bachelor’s | 40.000–59.999 | 48 | 20 | High | Online | 0 |
| P10 | 25-34 | Master’s | ≥ 80.000 | 60 | 55 | Medium | In-person | 13–24 |
| P11 | 35-44 | Bachelor’s | ≥ 80.000 | 62 | 35 | Low | Online | 7–12 |
| P12 | 18-24 | Associate | 22.501–39.999 | 46 | 20 | High | Online | 7–12 |
| P13 | 25-34 | PhD | 60.000–79.999 | 51 | 30 | High | In-person | 0 |
| P14 | 35-44 | Bachelor’s | 40.000–59.999 | 59 | 25 | Medium | In-person | 7–12 |
| P15 | 45-54 | High School | 40.000–59.999 | 56 | 30 | Low | Online | 1–6 |
| P16 | 18-24 | Bachelor’s | ≤ 22.500 | 47 | 35 | Medium | Online | 0 |
| P17 | 25-34 | Master’s | 60.000–79.999 | 55 | 40 | Medium | In-person | 7–12 |
| P18 | 25-34 | Bachelor’s | 60.000–79.999 | 52 | 30 | Low | Online | 1–6 |
| P19 | 18-24 | Bachelor’s | 22.501–39.999 | 49 | 45 | Medium | Online | 1–6 |
| P20 | 25-34 | Master’s | ≥ 80.000 | 64 | 60 | Low | In-person | 13–24 |
| P21 | 25-34 | Bachelor’s | ≥ 80.000 | 58 | 20 | High | Online | 0 |
| P22 | 35-44 | Bachelor’s | 40.000–59.999 | 53 | 30 | Medium | Online | 7–12 |
| P23 | 25-34 | Associate | 40.000–59.999 | 50 | 35 | Low | In-person | 13–24 |
| P24 | 25-34 | Master’s | 60.000–79.999 | 59 | 25 | High | Online | 0 |
| P25 | 35-44 | PhD | ≥ 80.000 | 62 | 30 | High | In-person | 13–24 |
| P26 | 35-44 | Bachelor’s | ≥ 80.000 | 61 | 25 | Low | Online | 7–12 |

**Table S5.** Semi‑structured in‑depth interview guide (researcher‑facing; anonymized)

| Module / Theme | Construct | Purpose (analytic focus) | Primary, non‑leading question | Deepening probes (use selectively) | Evidence to capture (coding anchors) | Confidentiality guardrails |
| --- | --- | --- | --- | --- | --- | --- |
| Opening & consent | — | Set scope, ensure consent, reduce evaluation anxiety | “Thanks for participating. We are interested in everyday mobility choices. May I confirm your consent to an audio recording and anonymized use of your answers?” | If hesitant: “We do not need names of people, streets, or brands—general descriptions are sufficient.” | Verbal consent; comfort level | No names of persons/streets/companies; no exact home/work addresses |
| Travel context | — | Situate typical trips; identify bottlenecks | “How do your typical weekday trips unfold from door to door?” | “Where do delays usually occur?” “What alternatives do you consider?” | First/last‑mile pain points; transfer frictions; weather effects | Avoid exact route names; ask “in your area/route type” |
| Utility & performance | PE | Time savings, reliability, access | “In what ways could an e‑scooter make your trips more predictable or efficient, if at all?” | “Where exactly would time be saved?” “On days when it doesn’t help, what changes?” | Time predictability; range adequacy; ride comfort; failure points | No specific employer/site names |
| Enjoyment vs utility | HM | Affective value without leading | “To what extent does enjoyment or fun matter relative to practical value in your decision?” | “Can enjoyment sustain use if utility is uneven?” | Hedonic cues as amplifier vs. driver | Keep neutral tone; no prestige/brand cues |
| Forward‑looking views | EF | Expectations on sustainability, availability, maturity | “How do you expect this mode to evolve in your city over the next 1–2 years?” | “Range/reliability/safety—what is likely to improve?” | Anticipated network growth; perceived environmental benefit | Avoid municipal identifiers; speak generally (“local authorities”) |
| Value & price salience | LPS / Value | Thresholds; total cost of ownership (TCO) | “When would the price feel justified for you?” | “What matters more: introductory price, warranty, or service continuity?” “What hidden costs worry you?” | Price thresholds; TCO factors (battery, parts, downtime) | No personal income figures; use ranges if needed |
| Autonomy (control) | PA | Schedule control; door proximity | “How might an e‑scooter change your control over when you leave/arrive?” | “Parking within a few meters—how important is that?” “Any rules that complicate it?” | “No waiting” narratives; door‑proximity; parking clarity | No mention of specific parking lots/buildings |
| Competence (self‑efficacy) | PC | Learning curve; safety routines | “What would make you feel fully confident operating one in everyday conditions?” | “Which micro‑skills matter (braking, positioning)?” “What helps confidence stick?” | Skill acquisition; safety habits; near‑miss reflections | No crash locations or third‑party identifiers |
| Exploration | IC | Innovative consumption (ancillary) | “How inclined are you to explore new features or routes once you feel comfortable?” | “What would you try first and why?” | Curiosity as trigger; feature exploration | Keep examples generic (no app brand names) |
| Ownership intent | PI | Triggers/conditions for purchase | “Under which conditions would purchase make sense for you?” | “Top three non‑negotiables?” “What would postpone the decision?” | Purchase criteria; risk trade‑offs | No store/retailer names |
| Infrastructure | Context | Lanes, surfaces, traffic norms | “How do lane protection and surface quality along your routes affect this choice?” | “Where does the flow ‘glide’ and where does it break?” | Protected links; surface roughness; conflict points | Describe types (“cobbled center”), not exact streets |
| Governance & parking | Context | Rules clarity/enforcement | “How clear are parking/usage rules from your perspective?” | “Any frictions or fines you have seen/heard about?” | Rule clarity; perceived fairness | No agency/person names |
| Service & warranty | Context | Downtime risk; continuity | “If a repair were needed, what turnaround would you consider acceptable?” | “Would a loaner/fast‑track service change your view?” | Warranty salience; acceptable downtime; service access | No shop names; keep generic |
| Theft & insurance | Context | Risk premium | “How do theft and insurance considerations factor into your thinking?” | “What protections would reduce that concern?” | Storage norms; insurance acceptability | No precise storage locations |
| Ownership vs rental | Context | Usage intensity; seasonality | “When would renting be preferable to owning?” | “How do weather/semester breaks affect this?” | Seasonal switching; usage intensity heuristics | No platform names |
| Multimodal fit | Context | Integration with transit | “How would it connect with your usual bus/metro segments?” | “Which transfer points would benefit most?” | First/last‑mile logic; transfer time cuts | No station names—say “transfer points” |
| Equity & affordability (optional) | Context | Perceived fairness | “How fair do pricing models feel across different users?” | “What would improve perceived fairness?” | Discount logic; student/low‑income sensitivity | No employer/institution identifiers |
| Closing & debrief | — | Summarize; invite final input; ethical close | “If you were to advise designers or policymakers in one sentence, what would you say?” | “Anything important we did not cover?” | Salient closing message | Thank participant; restate anonymization |

**Table S6.** Multicollinearity Assessment in the Structural Model

|  | PA | PC | EF | LPS | HM | PE | PI | IC |
| --- | --- | --- | --- | --- | --- | --- | --- | --- |
| PA |  |  |  |  |  |  | 1.419 |  |
| PC |  |  |  |  |  |  | 1.497 |  |
| EF |  |  |  |  |  | 1.669 | 1.552 |  |
| LPS |  |  |  |  |  | 1.139 | 1.457 |  |
| HM |  |  |  |  |  | 1.637 | 2.623 |  |
| PE | 1.000 | 1.000 |  |  |  |  | 1.548 | 1.000 |
| PI |  |  |  |  |  |  |  |  |
| IC |  |  |  |  |  |  | 1.399 |  |

**Table S7.** Detailed Codebook with Frequencies (N=26)

| Theme | Sub-theme | Code | Short Operational Definition | Linked Quant Path | Dir | n (26) | % | Mentions | M/P |
| --- | --- | --- | --- | --- | --- | --- | --- | --- | --- |
| T1 Function-first adoption (PE pivot) | Utility, time, reliability | T1C1 PE_TimeSaving | Commute/trip time savings attributable to scooter use | PE→PI; PE→PA/PC | + | 21 | 80.8 | 40 | 1.9 |
|  |  | T1C2 PE_Flexibility | Leave/arrive on demand; no fixed schedules | PE→PA; PE→PI | + | 16 | 61.5 | 24 | 1.5 |
|  |  | T1C3 PE_Reliability | Predictable, dependable travel outcomes | PE→PI; PE→PC | + | 14 | 53.8 | 20 | 1.4 |
|  | Trip fit & multimodal | T1C4 PE_MultimodalIntegration | Fits first/last-mile with transit | PE→PI | + | 13 | 50.0 | 19 | 1.5 |
|  |  | T1C5 PE_RouteFit | Typical routes suitable for scooter | PE→PI (context-moderated) | + | 12 | 46.2 | 18 | 1.5 |
|  | Utility, time, reliability | T1C6 PE_RangeAdequacy | Single-charge range adequate for daily use | PE→PI | + | 12 | 46.2 | 17 | 1.4 |
|  | Comfort & conditions | T1C7 PE_RideComfort | Suspension/tires smooth out bumps | PE→PI; PE→PC | + | 11 | 42.3 | 16 | 1.5 |
|  |  | T1C8 PE_WeatherSensitivity | Weather reduces usability | Context (boundary) | − | 15 | 57.7 | 22 | 1.5 |
| T2 Autonomy dividend (PA conduit) | Schedule independence | T2C1 PA_ScheduleControl | Control over departure/arrival; “not waiting” | PE→PA; PA→PI | + | 20 | 76.9 | 32 | 1.6 |
|  | Route control | T2C2 PA_RouteChoice | Choosing shortcuts/side streets | PE→PA; PA→PI | + | 18 | 69.2 | 29 | 1.6 |
|  | Transit independence | T2C3 PA_IndependenceFromTransit | Reduced dependence on bus/metro | PE→PA; PA→PI | + | 16 | 61.5 | 24 | 1.5 |
|  | Psych. autonomy & parking | T2C4 PA_Spontaneity | Last-minute/unplanned trip freedom | PE→PA; PA→PI | + | 14 | 53.8 | 21 | 1.5 |
|  |  | T2C5 PA_ParkingFreedom | Easy parking/locking near destination | PE→PA; PA→PI | + | 15 | 57.7 | 22 | 1.5 |
|  |  | T2C6 PA_PsychologicalFreedom | Felt freedom/control while traveling | PE→PA; PA→PI | + | 15 | 57.7 | 22 | 1.5 |
| T3 Competence ramp (PC strongest) | Learning curve | T3C1 PC_LearningCurve | From novice to comfortable in days/weeks | PE→PC; PC→PI | + | 19 | 73.1 | 33 | 1.7 |
|  |  | T3C2 PC_SkillAcquisition | Starting/braking/turning mastery | PE→PC; PC→PI | + | 18 | 69.2 | 29 | 1.6 |
|  |  | T3C3 PC_BalanceControl | Balance/posture confidence | PE→PC | + | 15 | 57.7 | 23 | 1.5 |
|  | Mastery in traffic | T3C4 PC_TrafficNegotiation | Confidence with cars/pedestrians | PE→PC; PC→PI | + | 22 | 84.6 | 39 | 1.8 |
|  | Confidence & safety | T3C5 PC_NightRidingConfidence | Confident riding at night | PE→PC; PC→PI | + | 13 | 50.0 | 20 | 1.5 |
|  |  | T3C6 PC_SelfEfficacyGrowth | Global “I can handle it” belief | PE→PC; PC→PI | + | 17 | 65.4 | 28 | 1.6 |
|  |  | T3C7 PC_SafetyPractices | Helmet/speed/route timing routines | PE→PC; PC→PI | + | 16 | 61.5 | 25 | 1.6 |
| T4 Price as threshold (value) | Value & WTP | T4C1 LPS_WTP_Performance | Pay more if clear performance gains | LPS→PE; PE→PI | + | 17 | 65.4 | 27 | 1.6 |
|  |  | T4C2 LPS_ThresholdEffect | Price okay if under personal limit | LPS→PE; PE→PI | + | 18 | 69.2 | 30 | 1.7 |
|  | Ownership & economics | T4C3 LPS_TotalCostOwnership | Battery, parts, depreciation calculus | LPS→PE (context) | ± | 14 | 53.8 | 21 | 1.5 |
|  |  | T4C4 LPS_PromoSensitivity | Discounts/installments reduce friction | LPS→PE (indirect) | + | 12 | 46.2 | 17 | 1.4 |
|  |  | T4C5 LPS_RentalVsOwnership | Rental/subscription vs buy | LPS→PE (context) | ± | 13 | 50.0 | 19 | 1.5 |
| T5 Innovation ≠ identity (IC boundary) | Novelty orientation | T5C1 IC_CuriosityNotDecisive | Novelty interest, not decisive | PE→IC; IC→PI (weak) | ± | 12 | 46.2 | 16 | 1.3 |
|  |  | T5C2 IC_GadgetAppealLimited | Gadget appeal downplayed | PE→IC; IC→PI (weak) | − | 10 | 38.5 | 13 | 1.3 |
|  | Social image | T5C3 IC_SocialImageAmbivalence | Ambivalence about “scooter person” | PE→IC; IC→PI (weak) | − | 11 | 42.3 | 15 | 1.4 |
|  |  | T5C4 IC_FunctionOverNovelty | Function/utility prioritized over novelty | IC non-mediation; PE/PA/PC dominate | + | 14 | 53.8 | 21 | 1.5 |
| T6 Context & safety constraints | Infrastructure & env. | T6C1 CT_InfrastructureAvailability | Safe lanes/routes available | Moderates PE→PA/PC | + | 24 | 92.3 | 44 | 1.8 |
|  |  | T6C2 CT_RoadSurfaceRisk | Potholes/cobbles raise risk | Moderates PE→PC; ↓PE→PI | − | 23 | 88.5 | 41 | 1.8 |
|  |  | T6C3 CT_TrafficAggressiveness | Aggressive traffic/pedestrians | Moderates PE→PA/PC→PI | − | 22 | 84.6 | 39 | 1.8 |
|  | Regulation & governance | T6C4 CT_RegulatoryClarity | Clear rules/signage/parking norms | Boundary for PE→PA | ± | 16 | 61.5 | 26 | 1.6 |
|  |  | T6C5 CT_EnforcementTrust | Trust in consistent enforcement | Context boundary | ± | 14 | 53.8 | 22 | 1.6 |
|  | Ecosystem & norms | T6C6 CT_ParkingSupply | Parking/locking points available | Moderates PA→PI | ± | 17 | 65.4 | 28 | 1.6 |
|  |  | T6C7 CT_TheftVandalismConcerns | Theft/vandalism worries | Context; reduces intention | − | 16 | 61.5 | 26 | 1.6 |
|  |  | T6C8 CT_ServiceRepairAccess | Service/repair/parts access | Context; supports PE | + | 15 | 57.7 | 24 | 1.6 |
|  |  | T6C9 CT_InsuranceAvailability | Insurance availability/affordability | ↓Risk → ↑PA/PC | + | 12 | 46.2 | 19 | 1.6 |
|  |  | T6C10 CT_SocialNorms | Stigma/acceptance, courtesy norms | Moderates PE→PA/PC→PI | ± | 21 | 80.8 | 37 | 1.8 |
|  |  | T6C11 CT_WeatherHazards | Rain/wind/heat as safety hazards | Dampens PE/PC effects | − | 20 | 76.9 | 34 | 1.7 |

**Note.** Dir = expected direction of effect (+ increase, − decrease, ± context-dependent). % = n/26 × 100. M/P = mentions per participant (Mentions ÷ n; rounded to one decimal). The table reports both coverage metrics (n, %) and intensity metrics (Mentions, M/P).
